# Distribution, mobility and exposure risk of rare earth elements, uranium and thorium in soils impacted by illegal cassiterite–monazite mining in the southeastern Amazon, Brazil

**DOI:** 10.1007/s10653-026-03447-7

**Published:** 2026-08-31

**Authors:** Antonia Andreyna Pereira Rodrigues, José Tasso Felix Guimarães, Gabriel Caixeta Martins, Luiza Santos Reis, Edna Santos de Souza, Roberto Dall’Agnol, Sílvio Junior Ramos

**Affiliations:** 1 Instituto Tecnológico Vale (Vale Technological Institute), Rua Boaventura da Silva 955, Nazaré, Belém, Pará 66055-090 Brazil; 2 Universidade Federal do Sul e Sudeste do Pará, Tv. Manoel dos Santos, 2-122, São Felix do Xingu, Pará 68380-000 Brazil; 3 Programa de Pós-Graduação em Geologia e Geoquímica, Instituto de Geociências, Universidade Federal do Pará, Rua Augusto Corrêa, 1, Belém, Pará 66075-110 Brazil

**Keywords:** Rare earth elements, Artisanal cassiterite–monazite mining, EDTA-extractable mobility, Geoaccumulation index, Hazard index, Amazon

## Abstract

Illegal cassiterite and monazite mining in the Taboca district of São Félix do Xingu, southeastern Amazonia, Brazil, reworks alluvial materials developed on the Paleoproterozoic Antônio Vicente Granite. This A-type granite is naturally rich in the REE-, Th- and U-bearing minerals monazite, xenotime and zircon, so mining further concentrates rare earth elements (REE), thorium and uranium whose concentrations are already raised by lithology. We sampled 130 surface horizons (34 native soils, 96 tailing residues) and analyzed them by chondrite-normalized REE patterns, mass-transfer coefficients against a niobium-tantalum composite, ethylenediaminetetraacetic-acid (EDTA) extraction, redundancy analysis and locally derived contamination index. Native soils carry two mineralogical signatures: light REE with thorium tracking detrital monazite, and heavy REE with yttrium tracking xenotime and zircon. Europium anomalies are strongly negative throughout. Tailings concentrate heavy-mineral phases while losing most of the soil reactivity. Organic matter drops from 2.15 to 0.54%, effective cation exchange capacity from 3.23 to 1.29 cmolc dm^−3^, and aluminum saturation rises from 2.85 to 16.35%. EDTA-extractable fractions of the heavy REE and yttrium climb above 24% (ytterbium 24.6%; lutetium 24.3%). The geoaccumulation index places residues in moderate-to-extreme classes for middle and heavy REE plus yttrium. The cumulative hazard index for non-radiological exposure crosses 1 for adult miners (1.0–3.7) and reaches 18–67 for child residents. Predicted-environmental versus no-effect concentration ratios in surface water exceed 1 for lanthanum, cerium, neodymium, thorium, yttrium, ytterbium and uranium, identifying these as monitoring priorities. A source-rock-aware baseline is essential to separate mining-derived contamination from naturally elevated geogenic backgrounds in tropical tin–rare-earth provinces.

**Supplementary Information:**

The online version contains supplementary material available at https://doi.org/10.1007/s10653-026-03447-7.

## Introduction

The main REE-bearing phases are monazite [(Ce,La,Nd,Th)PO_4_], xenotime [YPO_4_] and zircon [ZrSiO_4_], and the way each mineral takes up REE, Th and U is controlled by a few coupled substitutions. Monazite accommodates light REE and Th through the cheralite exchange 2REE^3⁺^ ↔ Ca^2⁺^ + Th^4⁺^ and the huttonite exchange REE^3⁺^ + P^5⁺^ ↔ Th^4⁺^ + Si^4⁺^, whereas in xenotime and zircon the heavy REE and Y replace one another (Y^3⁺^ ↔ HREE^3⁺^) and the tetravalent cations U, Th and Hf enter the Zr site (U^4⁺^, Th^4⁺^, Hf^4⁺^ ↔ Zr^4⁺^). These mechanisms are why light REE and Th travel with monazite while heavy REE, Y, Hf and U travel with xenotime and zircon (Förster, 1998a, b; Hoskin & Schaltegger, 2003; Spear & Pyle, 2002). Illegal mining is among the most pervasive and poorly regulated extractive activities in the Amazon Basin, driving deforestation, soil destruction, and metal mobilization across millions of hectares of the Amazon Basin, which spans Brazil, Peru, Bolivia, Colombia, Ecuador, Venezuela, Guyana, Suriname and French Guiana (Figueiredo et al., 2025; Salomão et al., 2023; Sonter et al., 2017; Veiga et al., 2006). In the Iriri-Xingu Domain of southeastern Amazonia, illegal mining activities target cassiterite and monazite hosted by the Paleoproterozoic Velho Guilherme Intrusive Suite (VGIS), an evolved A-type granite suite carrying accessory minerals that concentrate specific elements: monazite and xenotime (light and heavy REE, Th, U), zircon (Zr, Hf, U, Th and heavy REE), ilmenite (Ti, Fe) and cassiterite (Sn). Because these minerals are resistant to weathering, they accumulate in the residual and alluvial soils and raise the soil concentrations of REE, Th and U (Melo et al., 2021; Teixeira et al., 2005). This creates a critical geochemical assessment challenge, as gravity-based beneficiation selectively concentrates the same elements whose concentrations are already raised by lithologic control, complicating the distinction between mining derived contamination and the naturally extreme geochemical background of ore-proximal granitic soils (Reimann et al., 2005; Spear & Pyle, 2002).

Despite growing recognition that REE and actinide concentrations in soils affected by illegal mining may pose radiological and ecotoxicological risks (Hu et al., 2006; Ramos et al., 2016), environmental studies in the Amazon have focused predominantly on mercury in gold mining (Crespo-López et al., 2021) or other mining contexts (Sahoo et al., 2023). In tropical mineralized terrains naturally enriched in REE-bearing accessory minerals, the absence of lithology-constrained geochemical baselines complicates the distinction between geogenic enrichment and mining-related concentration processes. This gap is particularly critical in the Taboca district, São Félix do Xingu, Pará, where illegal cassiterite-monazite mining on soils derived from the Antônio Vicente Granite (AVG) has operated since the 1970s (Carvalho & Mello, 2024), and where recent socio-environmental pressures have intensified alluvial reworking and tailings dispersion (STF, 2024).

To address this gap, we analyzed 130 surface samples (34 native soils and 96 tailing residues) using three complementary approaches: (i) chondrite-normalized REE systematics and multi-element normalization to AVG lithotypes to establish lithogenic baselines and identify principal mineral hosts; (ii) mass-transfer coefficients (τ) referenced to GM(Nb,Ta) to quantify selective enrichment in tailing residues; and (iii) EDTA extraction combined with soil fertility parameters and redundancy analysis (RDA) to assess REE mobility and its edaphic controls. Contamination index (Igeo) was calculated using locally derived, median-based local thresholds rather than generic guidelines. The objectives of this study were: (1) to establish the first source-rock-constrained REE baseline for AVG soils; (2) to quantify element-selective enrichment in tailing residues; (3) to determine potentially mobile REE fractions and their relationship to mining-driven soil degradation; and (4) to provide a transferable framework for contamination assessment in tropical REE-Sn provinces with naturally heterogeneous REE backgrounds.

## Study area

### Socioenvironmental context and land use

**Fig. 1:**
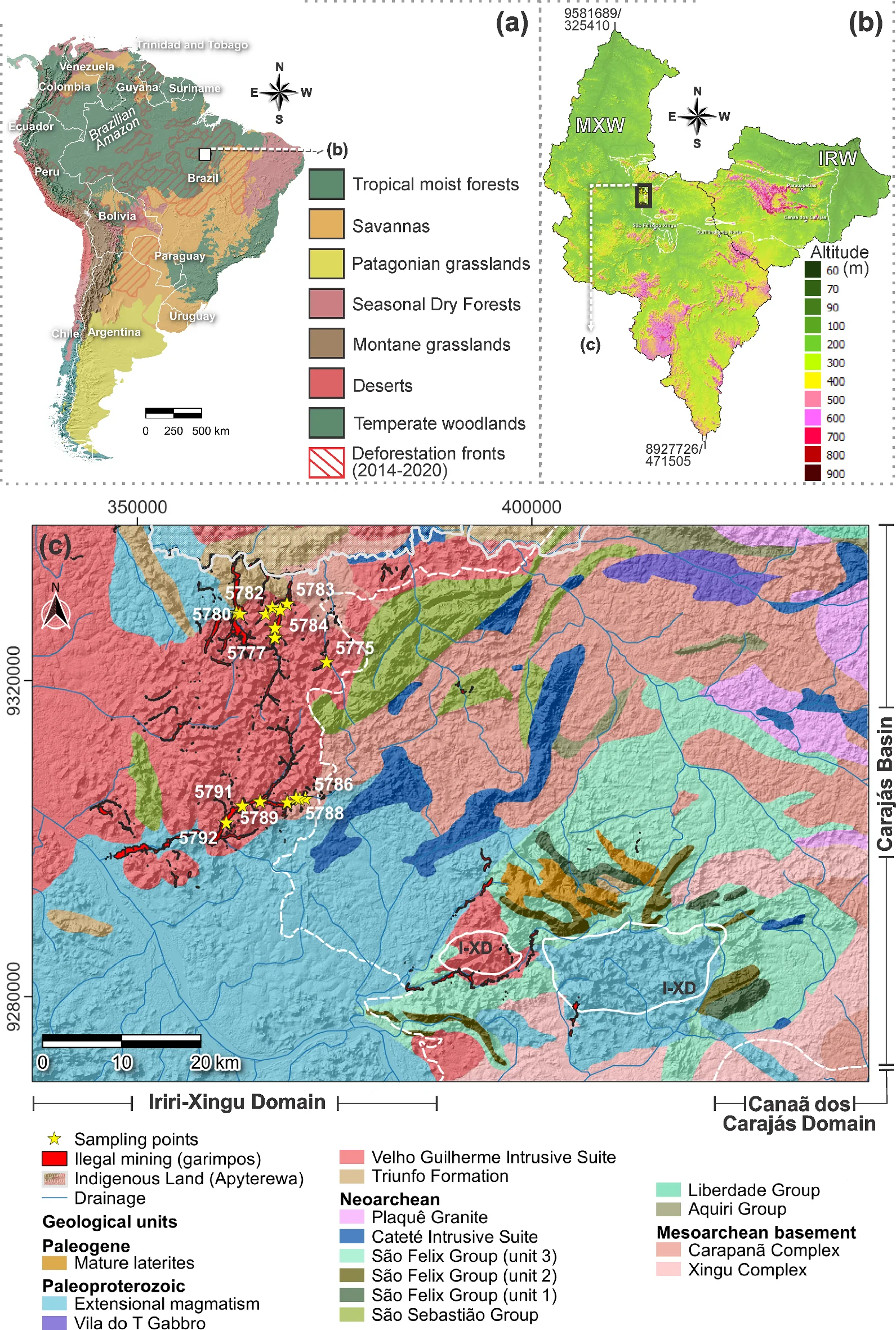
**a** Location of the study area within major South American biomes (adapted from Olson et al., 2001), overlaid with deforestation fronts from 2014 to 2020 (WWF, 2021). b hypsometric map of the Itacaiúnas River Watershed (IRW) and Middle Xingu River Watershed (MXW) in southeastern Amazonia, highlighting the Carajás Basin (dashed white line) within the Carajás Mineral Province (CMP) **c** Geological map of the study area (modified from Costa et al., 2016; Oliveira et al., 2022), showing sampling points (yellow stars) within zones of illegal alluvial cassiterite and monazite mining (red shading with black outline), specifically in the AVG site

The study was carried out in the Taboca district of São Félix do Xingu, in southeastern Amazonia, Brazil (Fig. 1a). The district (about 20,000 inhabitants) combines cattle ranching with informal and illegal mining of cassiterite, the tin ore, and monazite, a phosphate that carries Th, U and the REE (TRT-8, 2024). Mining started as a regulated cassiterite operation in the 1970s and closed in 1994; since 2011, unregulated garimpo has expanded and now dominates the reworking of the alluvial sediments and tailing residues that we sampled (Carvalho & Mello, 2024).

The area lies within the Apyterewa Indigenous Land (Fig. 1b), home to the Parakanã people, where a court-ordered removal of non-Indigenous occupants has been under way since 2023 and where deforestation dropped sharply in 2024 (FUNAI, 2024; MPI, 2024; STF, 2024). For our purposes, the point that matters is that this setting has pushed local activity toward the reworking of alluvial sediments and tailing residues along rivers and access tracks, which increases direct handling of, and exposure to, mining-derived wastes and disturbed soils.

### Climate and hydroclimate

São Félix do Xingu has an equatorial monsoonal climate (Am in the Köppen-Geiger system; Peel et al., 2007), with a wet season from December to May, annual rainfall of 1,800–2,500 mm and mean temperatures of 22–28 °C (INMET, 2024). This strong seasonality is what drives the wet-season remobilization and downstream transport of the alluvial sediments and mining residues studied here (Espinoza et al., 2019; Marengo et al., 2008).

### Geological framework of the Iriri-Xingu Domain, Carajás Basin, and Canaã dos Carajás Domain

The study area is in the western Iriri-Xingu Domain (I-XD), near its contacts with the Carajás Basin (CB) and the Canaã dos Carajás Domain (CCD) (Fig. 1b; Costa et al., 2016; Oliveira et al., 2022; Vasquez & Rosa-Costa, 2008). The CB and CCD basements are dominated by Mesoarchean granite-migmatite complexes, notably the Xingu and Carapanã complexes, later intruded by Neoarchean granitoids and mafic–ultramafic bodies (Barros et al., 2009; Dall’Agnol et al., 2017; Siepierski & Ferreira Filho, 2020). The CB also preserves Neoarchean metavolcano-sedimentary successions of the São Félix, São Sebastião, and Aquiri groups. During the Cretaceous to Paleogene, intense tropical weathering formed mature laterites on uplands such as Serra de Campos (Costa, 1991).

### Tin-REE mineralization in the Velho Guilherme Intrusive Suite and detrital pathways

Within this framework, the I-XD records Paleoproterozoic magmatism that includes localized sedimentary basins, felsic intrusions enriched in Sn of the Velho Guilherme Intrusive Suite (VGIS), phaneritic mafic bodies such as the Vila do T Gabbro, and associated volcanic units (Teixeira et al., 2005; Vasquez & Rosa-Costa, 2008). The VGIS comprises Paleoproterozoic anorogenic granites in the São Félix do Xingu region, notably the Antônio Vicente, Mocambo, Velho Guilherme, Bom Jardim, Serra da Queimada, Benedita, Ubim, and Rio Xingu massifs, which intrude Archean to Paleoproterozoic country rocks of the Rio Maria and Iriri-Xingu domains (Dall’Agnol et al., 1993; Teixeira et al., 2005; Dall’Agnol et al., 2005; Melo et al., 2021; Barros Neto et al., 2025). These plutons are dominantly metaluminous to peraluminous syenogranites and monzogranites with A-type (A2) affinity, shaped by strong fractional crystallization, and they record crystallization ages clustered at approximately 1.88 to 1.86 Ga (Teixeira et al., 2005, 2018). These granites belong to the Carajás Mineral (metallogenic) Province of the eastern Amazonian Craton (Dall’Agnol et al., 2005). Single-zircon Pb-evaporation ages place the emplacement of the Antônio Vicente, Mocambo and Rio Xingu massifs at about 1867–1862 Ma, and Nd model ages of 3.0–3.2 Ga with strongly negative εNd(t) point to Mesoarchean crustal sources with a long crustal residence, in line with their evolved, tin-fertile A-type (rapakivi) character (Teixeira et al., 2002, 2005, 2018).

Regionally, VGIS granites display chondrite-normalized gull-wing REE patterns, strong negative Eu anomalies, and low (Gd/Lu)_*N*_, which indicate relative HREE enrichment commonly linked to F-rich late magmatic liquids. The Antônio Vicente Granite (AVG) shares these first-order traits but it shows less pronounced Eu anomalies in less evolved facies, consistent with internal fractionation differences and variable hydrothermal overprint across the suite (Dall’Agnol et al., 1993; Teixeira et al., 2005). The Serra da Queimada Granite, also within the VGIS, consists of hololeucocratic to leucocratic syenogranites and monzogranites with A-type A2 signatures, peraluminous to weakly metaluminous and ferroan compositions, marked negative Eu anomalies in evolved facies, and Zr-saturation temperatures of about 870 to 754 °C (Melo et al., 2021).

Hydrothermal alteration and greisenization are widespread and host cassiterite and locally wolframite in veins and greisens (Dall’Agnol et al., 1993; Teixeira et al., 2005). Quartz CL and LA-ICP-MS studies in Mocambo and Velho Guilherme document the transition from magmatic to hydrothermal quartz and link late quartz generations to Sn mineralization. In the field, cassiterite occurs in greisenized rocks and quartz veins, and it is widely recovered from placer and alluvial deposits exploited by garimpos, from which tailing residues are generated, which provides a direct detrital source for alluvial Sn anomalies (Barros Neto et al., 2025).

### Link to sampling media and exposure context

The environmental compartments of interest in this study area are the alluvial sediments and the soils developed over, or derived from, the AVG of the VGIS. In tropical humid settings such as São Félix do Xingu, weathering, erosion, and hydraulic sorting concentrate detrital cassiterite and REE-bearing accessory phases such as monazite, zircon, and xenotime in valley-floor deposits and in the adjacent soils. Because the VGIS hosts Sn mineralization linked to evolved A-type granites and contains REE-Th-U accessory assemblages, a source-to-sink pathway that enriches Sn, U, Th, and REE in the surficial cover is the expected geochemical background against which any contribution from illegal and artisanal cassiterite–monazite mining must be evaluated.

## Material and methods

### Sampling strategy and analytical procedures

**Fig. 2:**
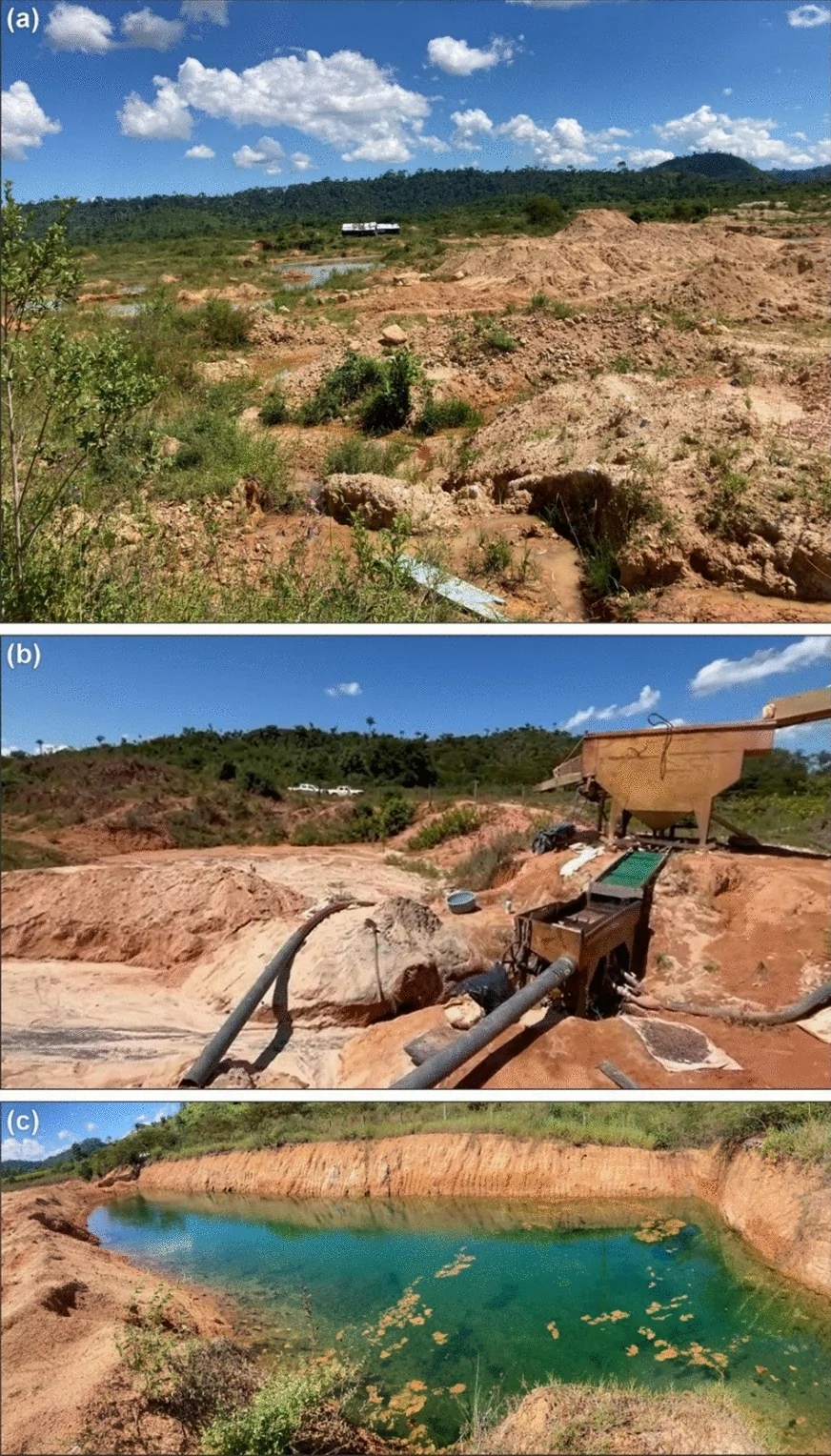
Illegal alluvial cassiterite and monazite mining in the study area. **a** Intensive reworking of alluvial sediments. **b** Artisanal separation of cassiterite and monazite using a hydraulic shaker and sequential sieving. **c** Abandoned dam with macroscopic signs of acid drainage, including greenish-blue water with in-situ pH ≤ 4 (portable pH meter), acidophilic algal growth along margins, absence of visible aquatic or riparian fauna, and a metallic odor

A minimum of five samples was collected for each geological unit, including the six dominant soil classes, ensuring both statistical and geospatial representativeness. The dominant soil classes encountered in the study area are Ultisols (Argissolos) and Inceptisols (Cambissolos) on the AVG terrain, with subordinate Entisols (Neossolos) and Oxisols (Latossolos) on uplands, and Fluvisols (Neossolos Flúvicos) and Gleysols (Gleissolos) in unaltered alluvial-valley settings (USDA Soil Taxonomy; SiBCS: Santos et al., 2018) equivalents in parentheses). In total, 130 surface samples were collected across the study area, comprising 34 native soils and 96 tailing residues derived from illegal cassiterite-monazite mining. Samples were collected from the 0–20 cm surface layer in areas influenced by active or abandoned illegal mining operations. Native soils were collected in areas with no visible mining disturbance but developed on, or directly derived from the granites of the AV batholith, whereas tailing residues were collected from reworked alluvial deposits and processing wastes associated with active or abandoned tailing sites. Figure 2 illustrates the field conditions observed in tailing-residues area, including reworked alluvial sediments (Fig. 2a), artisanal mineral separation (Fig. 2b), and an abandoned dam with signs of acid drainage (Fig. 2c).

In the laboratory, samples were air-dried, disaggregated and sieved to fine earth (Ø < 2 mm), then split and pulverized (SGS GEOSOL preparation routine PRS80J: drying, disaggregation, sieving at 80 mesh and pulverization of 300 g of the passing fraction), with a barren-quartz wash of the mill between samples (routine WSH78) to prevent carry-over. A tungsten-carbide mill was not used, which is consistent with the reported W, Nb and Ta (elements a tungsten-carbide vessel would contaminate) and with the W depletion documented in the Results. The geochemical evidence supporting the absence of W contamination is discussed in the Results. Major oxides were determined by X-ray fluorescence (XRF) on glass discs produced by lithium-tetraborate fusion (SGS GEOSOL method XRF79C), with loss on ignition (LOI) measured gravimetrically at 1000 °C (method PHY01E) and total carbon by combustion with infrared detection (method CSA17V). All whole-rock and pseudo-total analyses were performed at SGS GEOSOL Laboratórios (Vespasiano, Minas Gerais, Brazil; ISO 9001:2015 and ISO 14001:2015 certified). Trace elements, including REE+Y, U, Th, Sc and W, were quantified by inductively coupled plasma optical-emission and mass spectrometry (ICP-OES/ICP-MS) in two operational pools: (i) whole-material (total) concentrations after lithium-metaborate fusion (SGS GEOSOL method IMS95A), which dissolves refractory accessory minerals, with detection limits of about 0.05–0.1 mg kg^−1^ for the REE, Th and U; and (ii) pseudo-total concentrations after aqua regia digestion (SGS GEOSOL method ICM14B; ISO 11466:1995), the pool identified as “ICM14B” in the dataset, which operationally targets the aqua-regia-soluble fraction of soils. REE were measured on their least-interfered isotopes (e.g., ^139^La, ^140^Ce, ^141^Pr, ^146^Nd, ^147^Sm, ^153^Eu, ^157^Gd, ^159^Tb, ^163^Dy, ^165^Ho, ^166^Er, ^169^Tm, ^172^Yb, ^175^Lu, ^89^Y, ^45^Sc, ^238^U, ^232^Th), with oxide- and hydroxide-based isobaric interferences (notably light-REE and Ba oxides on the mid-to-heavy REE and on Eu) monitored and corrected under the laboratory’s ISO-accredited ICP-OES/ICP-MS procedure, oxide formation being kept low by instrument tuning (CeO⁺/Ce⁺ typically < 2–3%). The full instrumental parameters are held in the SGS GEOSOL method record and are available on request. Because aqua regia does not fully dissolve most silicates and its efficiency varies among matrices and elements (Supplementary Table 1), the aqua-regia results are treated throughout as pseudo-total rather than absolute totals (ISO 11466:1995).

Potentially extractable fractions were obtained by a one-step EDTA extraction at BIOAGRI Ambiental. EDTA is used here as a chelation-based operational pool that gives a standardized estimate of extractable forms rather than a formal geochemical speciation. Following the single-extraction approach for soils (Lihong et al., 1999; Loell et al., 2011), air-dried subsamples were shaken for 90 min with 0.025 mol L^−1^ EDTA at a fixed soil:solution ratio of 1:10 (mass:volume); the suspension was then centrifuged (≈513 relative centrifugal force) and the supernatant microfiltered through a 0.45 µm membrane, following the single-extraction protocol of Loell et al. (2011). The absolute sample mass and extractant volume follow the accredited BIOAGRI standard operating procedure (method 5706), and the extract analysed by inductively coupled plasma mass spectrometry (ICP-MS) at BIOAGRI Ambiental (Bioagri Laboratórios Ltda./Mérieux NutriSciences, Piracicaba, São Paulo, Brazil; internal method 5706), with a quantification limit of 0.5 µg kg^−1^ for every REE, Y, Sc, U and Th and results reported on a dry-weight basis. BIOAGRI is accredited to ISO/IEC 17025 by the Brazilian accreditation body CGCRE/INMETRO, so its determinations are traceable to internationally certified reference materials. Quality assurance for all analytical streams (XRF, total fusion, aqua regia and EDTA) included procedural blanks, replicate preparations and certified reference materials; the EDTA-extractable REE, Y, Sc, U and Th were quantified with a quantification limit of 0.5 µg kg^−1^ (BIOAGRI method 5706), and each result carried holding-time and sample-conformity quality flags on the certificate; each analytical batch at SGS GEOSOL carried preparation blanks, sample duplicates and replicates and control standards, and the per-element detection limits for the total (IMS95A) and aqua-regia (ICM14B) pools are compiled in Supplementary Table 4. Across 507 laboratory-replicate analyte comparisons in the GEOSOL certificates the median relative difference was 3.7% (72% of comparisons within 10% and 88% within 20%); the EDTA determinations at BIOAGRI (accredited to ISO/IEC 17025, which certifies analytical competence) are traceable to internationally certified reference materials, and the GEOSOL analyses (holding ISO 9001:2015 and ISO 14001:2015 quality- and environmental-management certification) were controlled in each batch with certified reference materials, procedural blanks and duplicates; the specific CRM identities and their recovery ranges are held in the laboratories’ batch quality-control records and are available on request.

Soil fertility parameters used as edaphic covariates (pH in water, exchangeable bases, potential acidity, CEC index, and related derived index), available P (Mehlich-1), and DTPA-extractable micronutrients were determined following the standardized Brazilian soil-testing protocols of the Embrapa Manual of Soil Analysis Methods (Teixeira et al., 2017); exchangeable Ca, Mg and Al were extracted with 1 mol L^−1^ KCl, exchangeable K and Na with Mehlich-1, and potential acidity (H+Al) with a pH 7.0 calcium-acetate buffer, all reported on a volume basis (cmolc dm^−3^) as is standard in Brazilian routine analysis. Mehlich-1 is the classical extractant introduced for acidic soils and remains widely used for routine soil P evaluation (Mehlich, 1953). DTPA-extractable Fe, Mn, Zn, and Cu were determined using the established DTPA soil test framework (Lindsay & Norvell, 1978). Particle-size fractions (total sand, silt, clay) were determined following standard soil physical analysis protocols and later treated as compositional data in the statistical workflow (Teixeira et al., 2017).

#### Statistical treatment of geochemical and edaphic data

Native soils (n = 34) and illegal mining tailing residues (n = 96) were summarized using robust statistics (median and interquartile range; Supplementary Table 2). Between-group differences for each parameter were evaluated using the Mann–Whitney U test (Mann & Whitney, 1947), and effect sizes were estimated using Cliff’s δ (Cliff, 1993). P-values were adjusted for false discovery rate using the Benjamini–Hochberg procedure (Benjamini & Hochberg, 1995). Soil pH was measured in a 1:2.5 soil:water suspension (not pore or surface water); organic carbon (OC) was determined by wet oxidation and organic matter (OM) estimated as OC × 1.724 (Teixeira et al., 2017).

REE (including Y), U, Th, and Sc were evaluated separately for the aqua regia (AR; pseudo-total) and EDTA (potentially extractable) fractions. Geochemical variables were log10-transformed to reduce skewness and the influence of extreme values. Grain-size fractions (sand, silt, and clay) were treated as compositional data (Aitchison, 1986) and represented using isometric log-ratio (ilr) coordinates, where ilr1 contrasts sand versus the fine fraction and ilr2 contrasts silt versus clay (Egozcue et al., 2003). Throughout, the light REE (LREE) are La, Ce, Pr, Nd, Sm and Eu, and the heavy REE (HREE) are Gd, Tb, Dy, Ho, Er, Tm, Yb and Lu; Y is grouped with the HREE because of its similar ionic radius and geochemical behaviour.

Environmental covariates used in the multivariate models comprised oxides determined by XRF (SiO_2_, Al_2_O_3_, Fe_2_O_3_, MnO, TiO_2_, and P_2_O_5_), pH (in H_2_O), organic carbon and organic matter (OC/OM), and the isometric log-ratio coordinates ilr1 and ilr2 that carry the texture information. In Redundancy Analysis the multi-element geochemistry is the response (dependent) matrix and the soil properties are the explanatory (predictor) matrix. RDA is a constrained ordination that extracts the fraction of the geochemical variation that can be linearly explained by the soil properties, so it quantifies how much of the REE+Y, U, Th and Sc signal is structured by the edaphic covariates. We therefore used the log_10_-transformed REE+Y, U, Th and Sc concentrations of each pool (aqua regia and EDTA) as the response matrix and the soil properties as predictors, fitting one RDA per pool (Legendre & Legendre, 2012). The joint significance of the constrained model and of the canonical axes was assessed by anova-like permutation tests (Legendre et al., 2011), and the significance of individual environmental vectors was evaluated with envfit under the same permutation scheme, adopting a threshold of *p* < 0.05. Group differences in multivariate geochemical composition were tested with PERMANOVA (Anderson, 2001), and homogeneity of multivariate dispersion was checked with PERMDISP (Anderson, 2006). All ordinations and permutation tests were run in R with the vegan package (Oksanen et al., 2022).

### Geochemical normalization, REE systematics, correlation, and mass-transfer analysis

To compare native soils with their lithologic sources and with tailing residues on a common scale, we used two complementary normalizations based on Li-metaborate dataset. First, element-by-element multi-normalization to representative Antônio Vicente Granite (AVG) lithotypes was applied to soils. Each soil was independently normalized to (i) a litho-average obtained from the pooled mean composition of the five less-evolved AVG varieties (BASMG, biotite–amphibole syeno- to monzogranite; BSGCl, biotite–syenogranite with chlorite; BMG, biotite–monzogranite; BSG, biotite–syenogranite; BSGA, altered biotite–syenogranite); and (ii) the BSGIA “Tin-rich” intensely altered facies, used as an ore-proximal reference. Because the sampled alluvial deposits and processing tailings are spatially associated with the most hydrothermally altered and mineralized AVG facies, BSGIA was adopted as the most representative ore-proximal normalizer; the litho-average reference is retained as a complementary lower-bound. Whole-rock compositions of the six AVG varieties used in this normalization are provided in Supplementary Table 3 (sheet ‘AVG_compositions’), compiled from Teixeira et al. (2005). These profiles were plotted on a log10 scale to resolve enrichments and depletions across majors and traces with different absolute magnitudes. Second, REE patterns were normalized to the Ivuna chondrite (Barrat et al., 2012) to assess inter-REE fractionation independent of absolute abundance. We report (La/Yb)_*N*_, (La/Lu)_*N*_, LREE/HREE, and (Y/Ho)_*N*_ (with Y grouped with the HREE throughout), together with index of Ce and Eu anomalies calculated according to Eq. 1 and Eqslongside the median in Supplementary Table 2, respectively:

1 $$\frac{Ce}{Ce}*=\frac{{Ce}_{N}}{\sqrt{{La}_{N}\times {Pr}_{N}}}$$

2 $$\frac{Eu}{Eu}*=\frac{{Eu}_{N}}{\sqrt{{Sm}_{N}\times {Gd}_{N}}}$$

CI-chondrite (Ivuna-type) normalizing values are from Barrat et al. (2012).

Because the native-soil dataset obtained by lithium-metaborate fusion departs from normality (Shapiro–Wilk test, *p* ≤ 0.05), we quantified bivariate associations among soil elements using Spearman’s rank correlation (ρ). We interpret ρ as an effect size: positive values indicate co-variation, negative values indicate inverse association, and larger absolute values |ρ| denote stronger monotonic relationships.

Mass‐transfer coefficients (τᵢ based on Li-metaborate digestion; Eq. 3) were computed after Brimhall and Dietrich (1987) using a high field strength elements (HFSE) composite reference to minimize host-mineral sorting biases in placer residues. We adopted geometric mean of Nb and Ta (GM(Nb,Ta)) as reference because the Nb/Ta ratio is the most stable between native soils and tailings (median shift +9.5%), whereas Hf/Ta and Hf/Nb shift by 84% and 64%, respectively, due to zircon retention. Sensitivity tests with Hf and GM(Hf,Nb,Ta) yielded consistent interpretations (reported in the Supplementary Material).

3 $$\tau i= \frac{{\left(\frac{Ci}{{C}_{\mathrm{G}\mathrm{M}\left(\mathrm{N}\mathrm{b},\mathrm{T}\mathrm{a}\right)}}\right)}_{residue}}{{\left(\frac{Ci}{{C}_{\mathrm{G}\mathrm{M}\left(\mathrm{N}\mathrm{b},\mathrm{T}\mathrm{a}\right)}}\right)}_{soil}}-1$$

### Contamination assessment and operational mobility index

To evaluate contamination in a region naturally enriched in REE due to lithogenic signatures, contamination and operational mobility index were calculated using pseudo-total and EDTA-extractable concentrations.

The geoaccumulation index (Igeo) was used to assess geochemical enrichment relative to a local background (Müller, 1969). Instead of generic regulatory thresholds, we used a locally derived background defined as the median concentration of the native-soil population for each element (Supplementary Table 2). This median-based baseline accounts for the naturally elevated REE, Th and U of VGIS-derived soils, and its robust dispersion is summarized by the modified median absolute deviation (mMAD = 1.4826 × MAD), reported alongside the median in Supplementary Tables 2 and 3. Two caveats apply. First, the Igeo was originally formulated by Müller (1969) for the < 2 µm clay fraction, whereas our samples are bulk fine earth (< 2 mm). The wider and more variable grain-size range increases the spread of reactive surface area, so absolute Igeo values and Müller’s descriptive classes should be read as relative, screening-level indicators rather than as strict clay-referenced categories. Second, because the baseline is the native-soil median, a sample equal to that median has Igeo = log2(1/1.5) ≈ −0.585 rather than zero, so the native soils cluster near −0.585 by construction and the Igeo is informative mainly for the tailing residues. Igeo was calculated according to Eq. 4: We also verified the classification against a Reimann-style robust anomaly threshold (median + 2 × mMAD; Reimann et al., 2014, 2018); the outcome is reported in Supplementary Table 3.

4 $$Igeo={\mathrm{log}}_{2}\left(\frac{{C}_{sample}}{1.5\times {C}_{baseline}}\right)$$

where C_sample_ corresponds to the concentration of the element in the soil sample and C_baseline_ represents the local geochemical baseline value. The 1.5 factor compensates for natural lithologic variability. Igeo values were classified as follows: Igeo ≤ 0, uncontaminated; 0 < Igeo ≤ 1, uncontaminated to moderately contaminated; 1 < Igeo ≤ 2, moderately contaminated; 2 < Igeo ≤ 3, moderately to heavily contaminated; 3 < Igeo ≤ 4, heavily contaminated; 4 < Igeo ≤ 5, heavily to extremely contaminated; and Igeo > 5, extremely contaminated (Müller, 1969).

To estimate the relative extractable fraction of REE, an EDTA-adjusted operational mobility index was calculated as the ratio between EDTA-extractable and pseudo-total concentrations multiplied by 100 (Niesiobędzka, 2023). This operational approach follows mobility index commonly applied to potentially toxic elements in soils and was adapted here to evaluate the relative mobility of REE in native soils and illegal tailing residues.

Building on the operational mobility index of Niesiobędzka (2023), three complementary indicators were derived from the EDTA/AR dataset to translate the geochemistry into screening-level human-exposure and ecotoxicological metrics. The EDTA/AR ratio is treated throughout this work as an operationally mobilizable fraction, not as a formal geochemical speciation, and not as a Risk Assessment Code (RAC) in the regulatory sense; the class boundaries (very low, < 5%; low, 5–10%; moderate, 10–20%; high, 20–40%; very high, > 40%) describe the mobility potential of each element under the standardized EDTA extraction protocol. (i) The Hazard Quotient (HQ) is the ratio of the average daily dose (ADD, mg kg^−1^ d^−1^) to the reference dose (RfD, mg kg^−1^ d^−1^): HQ_i_ = ADD_i_/RfD_i_. It was computed for soil ingestion, dust inhalation and dermal contact under two exposure scenarios (adult artisanal miner and child resident) following the US-EPA framework (US-EPA, 1989, 1996); oral RfD values for the REE were taken from Pang et al. (2002), and the US-EPA IRIS database was used for U and Th. The cumulative Hazard Index HI = Σ HQ_i_ was reported. (ii) The aquatic ecotoxicological screening PEC/PNEC was estimated from the EDTA-extractable concentrations as an operational proxy for the potentially mobilizable pool under chelating conditions, using literature soil–water Kd values (Mihajlovic & Rinklebe, 2018; Sholkovitz, 1995) and freshwater PNEC values compiled by Sneller et al. (2000) and González et al. (2014); the U threshold followed the British Columbia Ministry of Environment aquatic-life guideline (8.5 µg L^−1^) and Th was set at 1 µg L^−1^ as a conservative reference. Because soil–water Kd in tropical acidic systems spans roughly one order of magnitude (Mihajlovic & Rinklebe, 2018), PEC/PNEC values are interpreted as relative priorities and not as absolute thresholds. (iii) Data-quality screening included substitution of below-detection-limit values by LD/2 (Helsel, 2005), removal of two samples (5782-P4 and 5782-P7) flagged as analytically invalid in the XRF stream, and conservative masking of 22 sample-element pairs (0.9% of the dataset) in which the EDTA concentration exceeded the AR concentration, which is incompatible with the operational definition of EDTA as a subfraction of the pseudo-total pool.

## Results and discussion

### Multi-normalization and REE systematics to analyze native soils relative to their lithologic source

Native soils were collected on the AVG, an A-type granite batholith where late extensive fractionation and F-rich fluids generated evolved leucogranites with associated Sn-bearing greisens and a characteristic accessory assemblage documented for the AVG by Teixeira et al. (2005) that includes zircon, monazite, xenotime, ilmenite, titanite, apatite, cassiterite and, locally, wolframite; this assemblage is the mineralogical reference against which we interpret the soil geochemistry, rather than a direct mineralogical identification in our samples.

**Fig. 3:**
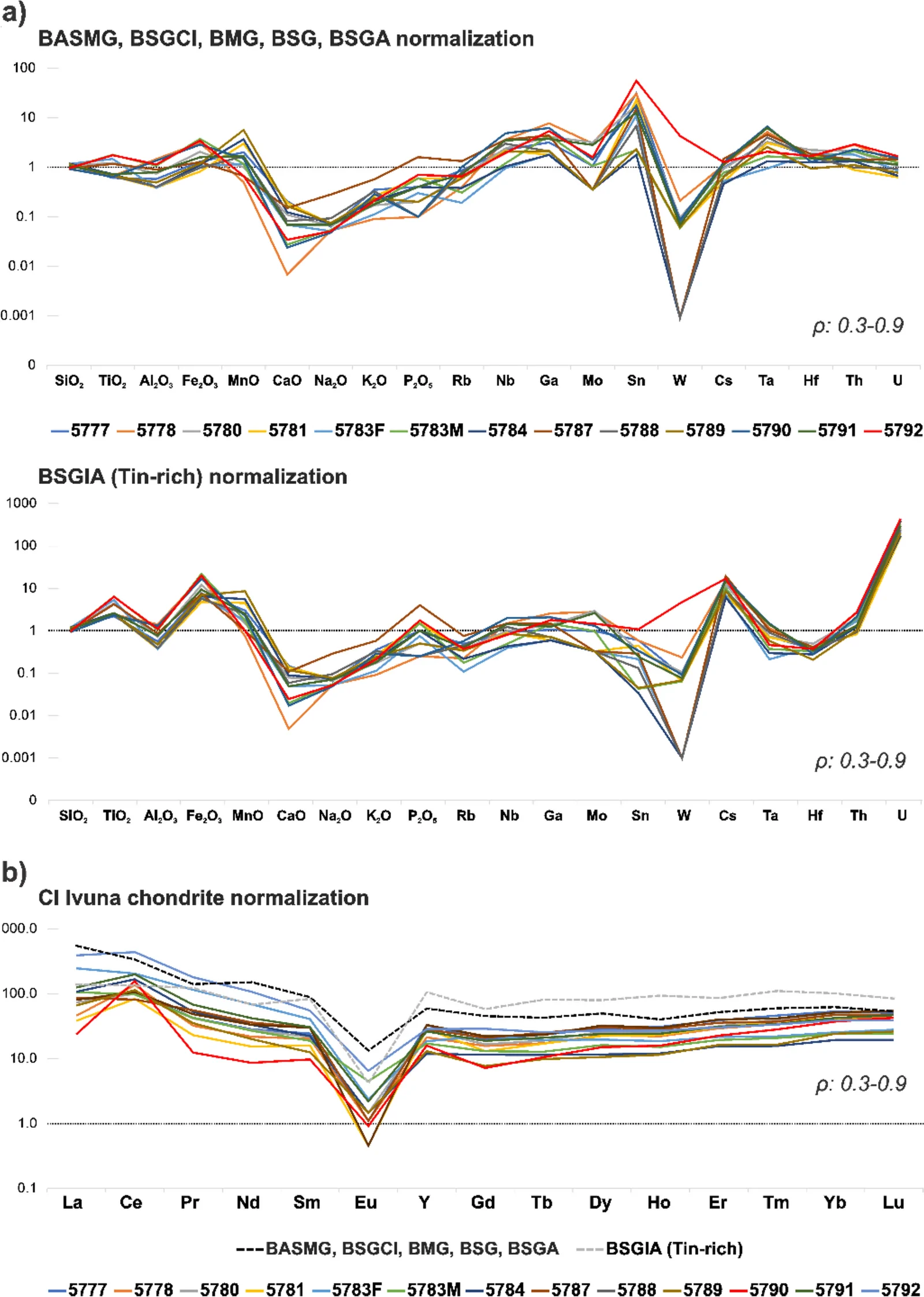
Major–trace element patterns for native soils of the AVG normalized: **a** to pooled AVG granites (BASMG, BSGCl, BMG, BSG, BSGA; top), and **b** to Tin-rich extremely evolved BSGIA (middle). Note the W trough in every soil except 5792 and Sn near BSGIA only in 5792; U > 1 vs BSGIA while Th ~ 1. **c** Ivuna chondrite–normalized REE (Barrat et al., 2012) for soils versus granite envelopes (dashed). HREE sum includes Y [HREE = Σ(Y, Gd–Lu)]; LREE = Σ(La–Sm)]. Rock abbreviations: BASMG, biotite–amphibole syeno- to monzogranite; BSGCl, biotite syenogranite with chlorite; BMG, biotite monzogranite; BSG, biotite syenogranite; BSGA, altered biotite syenogranite; BSGIA, intensely altered biotite syenogranite (Tin-rich). ρ values (minimum–maximum) are the pairwise Spearman correlations across all samples; 0.3–0.9: moderate to strong positive correlation

When normalized to the average composition of the main varieties of the AVG granites (BASMG, BSGCl, BMG, BSG, BSGA; whole rock analyses of Teixeira et al., 2005), all soils show strong Na_2_O, and CaO and moderate P_2_O_5_, K_2_O, and Rb depletions with low MnO positive anomalies, and near-unity Nb, Ga, Cs, Ta, Hf, Th, and U. It is observed systematically that Cs > Rb (Fig. 3a), an expected signature indicative of intense tropical weathering, with Cs fixation at the frayed-edge sites of weathered micas and illite (from biotite/muscovite alteration) and sorption to Fe-oxides and clays (Cornell & Schwertmann, 2003; Sawhney, 1972). Relative to the AVG source, W is strongly depleted in almost every soil (a deep negative trough in the AVG-normalized profile, i.e., soils contain much less W than the granite), except sample 5792. This depletion is consistent with tungstate mobility and with only limited detrital wolframite under oxidizing weathering (Bednar et al., 2008), and it is the opposite of what mechanical contamination from a tungsten-carbide mill would produce (which would add W, and also Co and Ta, raising them above the source). The stability of the Nb/Ta ratio between soils and residues (median shift + 9.5%; see the mass-transfer analysis) further argues against Ta (hence WC–Co–Ta) contamination, and W co-varies with Mo and Fe_2_O_3_ rather than showing a flat matrix-independent offset, so the low W values are geogenic. This low-W character of the AVG is itself expected from the reduced nature of these tin-fertile granites, which favours Sn over W enrichment, the latter occurring preferentially in oxidized A-type granites (Dall’Agnol et al., 2005). Using BSGIA (Tin-rich extremely evolved leucogranite) as the normalizer tightens dispersion (Fig. 3a, middle). Sn is slightly below BSGIA in most soils, whereas sample 5792 plots close to BSGIA for Sn and shows a mildly positive W, indicating local enrichment of cassiterite and possibly wolframite (Melo et al., 2021; Teixeira et al., 2005). Uranium exceeds unity relative to BSGIA, whereas Th remains near 1, a pattern typical of oxidative U(VI) mobilization followed by secondary retention on oxide/clay surfaces (Duff et al., 2002; Langmuir, 1978).

Soil REE patterns are LREE-enriched with flat to gently rising HREE segments and pronounced negative Eu anomalies. The chondrite-normalized (La/Yb)_*N*_ ratio, a petrological and geochemical indicator of REE pattern slope analogous to Eu/Eu*, varies between 0.64 and 9.97. These relatively low values are consistent with the strongly differentiated, A-type evolved character of VGIS granites, where late-magmatic F-rich fluids and intense fractionation typically produce flat to gently HREE-enriched patterns (Eby, 1992; Teixeira et al., 2005), paralleling the AVG envelopes (Fig. 3b). Eu/Eu* is uniformly low (0.02–0.29; averages of pooled granites ~ 0.21, BSGIA ~ 0.06; Teixeira et al., 2005), recording the feldspar-fractionated, evolved A-type signature of the parental granites (Eby, 1992; Teixeira et al., 2005). Ce/Ce* is > 1 in all soils, evidencing strong sequestration of Ce with oxidation of Ce(III) to Ce(IV)and scavenging, and ranges from mild to very strong with the peak at sample 5790 (Table 1). Sample 5790 also shows HREE-tilted pattern (La/Yb)_*N*_ = 0.64), consistent with xenotime/zircon control of the HREE tail (Bau & Dulski, 1996). By contrast, 5792 is the most LREE-skewed sample (La/Yb)_*N*_ = 9.97; LREE/HREE = 6.44; LREE = 437.5; Table 1), best explained by detrital monazite concentration (Melo et al., 2021; Teixeira et al., 2005). Across the dataset, LREE/HREE ranges from 1.13 to 6.44. Note that some samples (e.g., 5790 and 5781) have HREE-tilted patterns as indicated by (La/Yb)_*N*_ < 1. Yet LREE/HREE ratios are > 1 in the sums because Y is included in HREE, a useful distinction between shape-based index and sum-based budgets.

**Table 1:** Ce/Ce*, Eu/Eu*, (La/Yb)_*N*_,(La/Lu)_*N*_, LREE, HREE, LREE/HREE, and U/Th for native soils and representative rocks of the Antônio Vicente Granite (AVG)

Sample	Ce/Ce_*_	Eu/Eu_*_	La/Yb_*N*_	La/Lu_*N*_	LREE	HREE	LREE/HREE	U/Th
5777	1.80	0.05	1.52	1.59	104.88	77.10	1.36	0.83
5778	3.53	0.06	1.13	1.07	110.91	53.56	2.07	0.43
5780	1.81	0.08	1.54	1.56	99.12	65.33	1.52	0.49
5781	2.80	0.03	0.92	0.81	72.20	63.63	1.13	0.71
5783F	1.21	0.08	9.65	8.80	232.69	45.98	5.06	0.47
5783 M	1.45	0.29	4.42	4.41	104.48	40.04	2.61	0.58
5784	2.21	0.09	5.53	5.52	151.92	29.43	5.16	0.51
5787	1.18	0.04	1.81	1.80	96.75	69.16	1.40	1.04
5788	1.70	0.02	1.56	1.49	108.78	78.31	1.39	0.95
5789	2.36	0.15	2.72	2.53	100.55	31.00	3.24	0.70
5790	9.08	0.11	0.64	0.56	108.92	40.08	2.72	0.66
5791	2.16	0.09	2.94	2.67	182.60	62.47	2.92	0.81
5792	1.65	0.16	9.97	10.03	437.53	67.91	6.44	0.57
BASMG, BSGCl, BMG, BSG, BSGA	1.21	0.21	8.77	10.17	433.47	129.76	3.34	0.36
BSGIA	1.03	0.06	1.37	1.64	170.78	222.80	0.77	0.00

All REE parameters are CI Ivuna–normalized; subscript N denotes chondrite-normalized concentrations. HREE sum includes Y [HREE = Σ(Y, Gd–Lu)]; LREE = Σ(La–Sm). BSGIA U/Th = 0.00 reflects U < LOD. Rock abbreviations: BASMG , biotite–amphibole syeno- to monzogranite; BSGCl, biotite syenogranite with chlorite; BMG, biotite monzogranite; BSG, biotite syenogranite; BSGA, altered biotite syenogranite; BSGIA, intensely altered biotite syenogranite (Tin-rich)

**Fig. 4:**
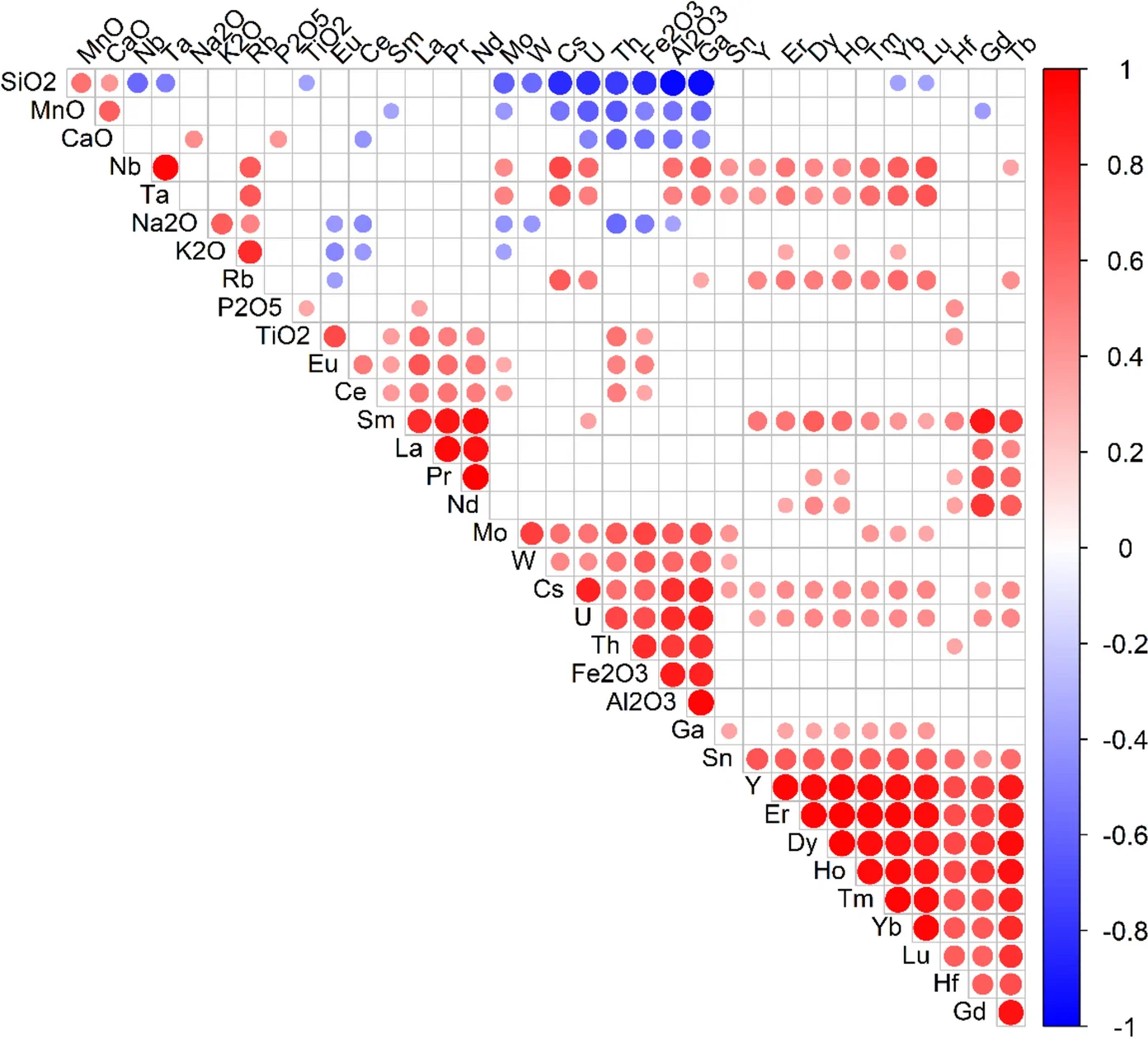
Spearman correlation matrix for native soils derived from the granite varieties of the Antônio Vicente Granite batholith (AVG). Pairwise Spearman rank coefficients (ρ) are shown for major oxides, trace elements, and REE (Y grouped with the HREE). Data are from lithium-metaborate fusion. The plot is upper-triangular. Each bubble’s color encodes the sign and magnitude of ρ on the right (blue: negative; red: positive) and area is proportional to |ρ|. Blank cells indicate coefficients near zero

The native soils are derived from the granite varieties of the Antônio Vicente batholith (AVG), and their covariance patterns mirror the heavy-accessory assemblage documented for those evolved A-type granites (Teixeira et al., 2005). The Spearman matrix resolves the REE into two coherent groups (Fig. 4). Light REE vary together and correlate with Th and, more moderately, with P_2_O_5_, consistent with control by monazite (Teixeira et al., 2005). Because monazite hosts trivalent LREE on the same lattice site, La, Pr, Nd, and Sm also vary coherently. Heavy REE and Y covary with Hf and Zr, which is the signature of zircon acting with xenotime (Teixeira et al., 2005). Nb and Ta show near-parallel behavior, consistent with a columbite-group mineral as the likely Nb–Ta host, although this phase was inferred from the element covariance rather than identified directly in our samples (Teixeira et al., 2005). Sn increases with Hf, fitting detrital cassiterite concentrated in the same heavy fraction as zircon. W follows Mo and Fe_2_O_3_, an expected pattern where wolframite occurs within oxide-rich microenvironments. TiO_2_ shows positive links to LREE and Hf, tying ilmenite and rutile to the hydraulically concentrated heavy suite. SiO_2_ follows an opposite trend: where quartz is high, most metals, both REE groups, Th, U, Fe_2_O_3_, and TiO_2_ are lower, recording quartz dilution relative to heavies and fines. Therefore, these relationships show that the soils preserve the mineral imprint of the AVG and provide a baseline for quantifying gains and losses by comparing soils with tailing residues.

### Element mass transfer (τ) from native soils to tailing residues

Building on the rock to soil alignment documented previously, where chondrite-normalized REE patterns and multi-normalizations showed that native soils inherit their geochemical template from evolved A-type granites of the AVG, we extend the provenance analysis to the soil to residue comparison using mass-transfer functions τ computed on total-element data for both matrices obtained by Li-metaborate digestion. This digestion for soils and residues removes reagent selectivity and yields element ratios that are directly comparable across hosts, so the τ results isolate process effects along the tailing line from the source signal that is already carried from bedrock to soil (Brimhall & Dietrich, 1987). To minimize artifacts from a single immobile denominator in a placer setting, we normalize to the geometric mean of Nb and Ta, GM(Nb,Ta). This composite HFSE reference is less susceptible to zircon control than an Hf-only choice and avoids the upward or downward skew observed when Ta or Nb are taken alone. Zircon is a dense and refractory carrier of Hf that is commonly retained in tailings, so an Hf-only denominator would depress τ for other enriched constituents wherever zircon leaks into residues (Hoskin & Schaltegger, 2003).

**Fig. 5:**
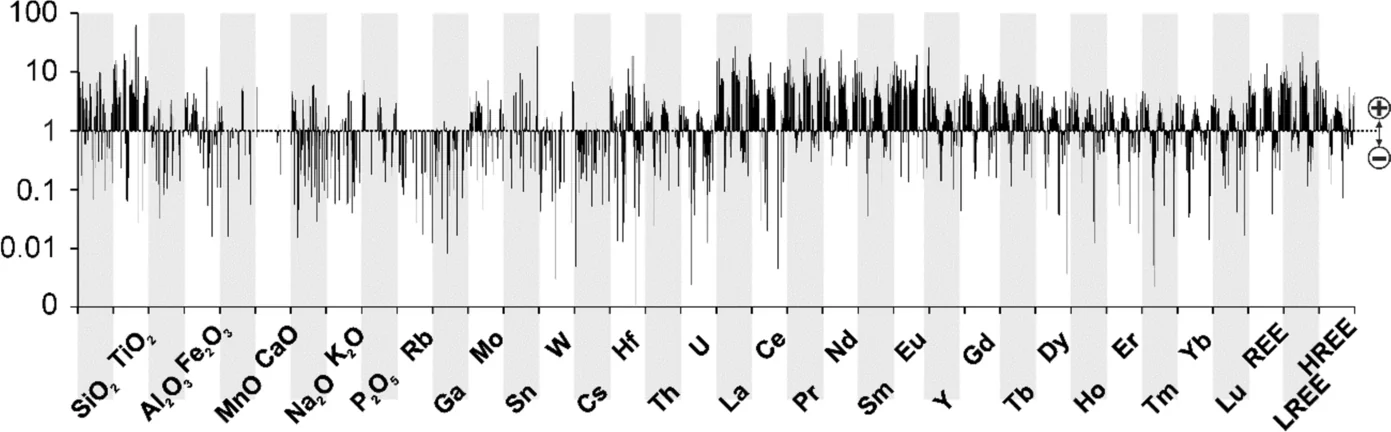
Log-scale mass-transfer coefficients for residues relative to native soils, calculated from total-element analyses by lithium-metaborate fusion and using GM(Nb,Ta) as the immobile reference. The horizontal dotted line at 1 corresponds to τ_i_ = 0 (no net gain or loss); values above 1 indicate enrichment in residues and values below 1 indicate depletion. Each vertical swarm shows the site-to-site spread. Original concentrations used to compute τ_i_ were measured as wt% for major oxides and mg kg^−1^ for trace elements; τ_i_ is unitless after normalization

The τ profiles show that SiO_2_ is mostly positive (Fig. 5), which indicates persistent quartz carryover into residues because gravity circuits tuned for dense cassiterite do not consistently purge coarse, light quartz. TiO_2_ is strongly positive with a long upper tail, which fingerprints retention of ilmenite, rutile and anatase, a consequence of cut size, residence time, or downstream scavengers not tuned to remove non-Sn heavies (Nzeh et al., 2023; Van Gosen et al., 2014). In contrast, Al_2_O_3_ is mostly negative and Na_2_O is also mostly negative but with a modest positive tail, which is consistent with preferential removal of Al–silicate fines such as kaolinite, gibbsite, and feldspar from the tailings line under dominant conditions, punctuated by episodes of fines co-settling when hydraulic or flocculation settings trap feldspathic material. Fe_2_O_3_ is generally positive, indicating in-pond formation and retention of ferric oxyhydroxides under oxidizing conditions. These freshly formed phases exert first-order control on trace-element partitioning by adsorption and co-precipitation and therefore matter directly for actinide and oxyanion behavior (Cornell & Schwertmann, 2003).

P_2_O_5_ is near neutral on average but exhibits a right-skewed distribution with a subset of markedly elevated values (Fig. 5). This distribution is diagnostic of spatially variable monazite retention in the residues because monazite is a rare-earth phosphate and the principal LREE host in this system. A subordinate contribution from detrital apatite (a minor accessory phase reported in the AVG; Teixeira et al., 2005) cannot be excluded, although the strong covariance between P_2_O_5_, LREE and Th is most parsimoniously explained by monazite. Where the flow path concentrates heavy accessories, residues show coincident increases in P_2_O_5_, LREE, and commonly Th, whereas samples with more efficient hydraulic rejection of heavy accessories remain near neutral in P_2_O_5_ and display lower τ for LREE and Th. Because both matrices were totally digested, positive τ for P_2_O_5_ records actual mass addition of phosphate-bearing solids rather than differential extraction, and the alignment of P_2_O_5_ with LREE and Th provides an internal provenance check that complements the REE systematics established earlier (Förster, 1998a, 1998b; Spear & Pyle, 2002).

Sn is heterogeneous, with many samples near neutral and episodic high positive excursions, which is the signature of variable cassiterite recovery related to fines bypass or short residence time. The site-to-site scatter also mirrors the uneven regional distribution of Sn mineralization across the sampled catchments, so operational selectivity and geological source strength both contribute and are kept distinct in our interpretation. W is commonly negative, which points to almost complete wolframite retention in the treatment process. Across the REE block the residues are systematically enriched with LREE exceeding HREE. This LREE-over-HREE enrichment reflects the dominance of monazite over xenotime and zircon and is carried from rock to soil to residue, in agreement with the monazite-controlled LREE budget described by Spear and Pyle (2002).

Actinide and oxyanion behavior integrate provenance hosts with surface processes documented in tailings waters. Th and U are mixed rather than uniformly positive. Thorium primarily follows monazite hydraulics (Williams et al., 2022). Samples that show strong additions of LREE and P_2_O_5_ tend to exhibit positive τ for Th, whereas improved monazite retention yields neutral or negative τ for Th. U reflects a competition between mineral association including xenotime and zircon and adsorption or co-precipitation as U(VI) on freshly precipitated Fe-oxides (Massey et al., 2014). U(VI) adsorption is efficient in acidic to circum-neutral, oxidizing waters and weakens in more alkaline or carbonate-rich conditions where uranyl-carbonate complexes dominate, producing the observed mix of positive and negative τ for U (Duff et al., 2002; Waite et al., 1994; Winstanley et al., 2019).

Mo is mixed positive and negative, which is diagnostic of molybdate’s pH- and competitor-dependent affinity for ferrihydrite and goethite. Sorption is strong at acidic to circum-neutral pH and declines at higher pH and in the presence of competing anions such as phosphate, conditions that vary across samples and over operational time in tailings impoundments (Brinză et al., 2008; Goldberg et al., 1996). Where individual REE patterns are interpreted, Ce anomalies should be considered within a redox-scavenging framework. Oxidation of Ce(III) to Ce(IV) and preferential uptake by Mn and Fe oxides can generate positive or negative anomalies depending on redox and ligand availability, including microbially mediated pathways, and this is consistent with the Fe-oxide signatures observed in the residues (Bau & Dulski, 1996; Moffett, 1990; Verplanck et al., 2004).

### Native soils versus tailing residues: fertility and texture shift

Native soils exhibit higher fertility and buffering capacity than tailing residues, whereas the residues behave as a coarse, low-retention discontinuous surficial cover after alluvial reworking (Table 2). Differences in trace-element and REE concentrations between native soils and tailing residues are also evident in the Aqua regia data (Supplementary Fig. 1).

**Table 2:** Summary of soil fertility and texture parameters for Antônio Vicente Granite (AVG) native soils (n = 34) and tailing residues (n = 96). Values are median (interquartile range)

Parameter	Native soils median (IQR)	Tailing residues median (IQR)	q (FDR)	Cliff’s δ	Effect
Organic matter (OM, %)	2.155 (1.34–2.45)	0.54 (0.48–0.623)	1.11e−13	−0.897	Large
pH (in H_2_O)	5.3 (5.1–5.8)	4.9 (4.8–5.1)	3.46e−05	−0.491	Large
CEC potential (T, cmolc dm^−3^)	6.405 (5.26–7.45)	2.465 (1.83–3.61)	1.28e−12	−0.849	Large
CEC effective (t, cmolc dm^−3^)	3.225 (2.51–4.09)	1.29 (0.98–1.87)	2.38e−13	−0.883	Large
Ca (cmolc dm^−3^)	2.52 (1.67–2.94)	0.73 (0.64–0.94)	6.80e−13	−0.864	Large
Mg (cmolc dm^−3^)	0.33 (0.242–0.477)	0.1 (0.07–0.19)	6.71e−08	−0.642	Large
K (mg dm^−3^)	93.35 (61.3–130)	30.65 (18.6–44.3)	3.36e−12	−0.83	Large
Na (mg dm^−3^)	5.98 (5.59–6.53)	5.345 (4.88–6.15)	0.0007	−0.41	Medium
Sum of bases (SB, cmolc dm^−3^)	3.13 (1.96–4.03)	0.95 (0.8–1.25)	1.28e−12	−0.851	Large
Base saturation (V, %)	50.25 (36.7–63.6)	43.35 (35.1–47.5)	0.0055	−0.335	Medium
P Mehlich-1 (mg dm^−3^)	6.9 (4.6–10.8)	3.4 (1.77–6.15)	2.45e−05	−0.505	Large
S (mg dm^−3^)	4.03 (3.09–5.11)	4.865 (2.95–8.28)	0.314	0.122	Negligible
N total (%)	0.181 (0.14–0.228)	0.029 (0.0191–0.043)	9.03e−15	−0.95	Large
Cu (DTPA; mg dm^−3^)	1.1 (0.625–1.85)	0.35 (0.2–0.7)	2.69e−07	−0.608	Large
Fe (DTPA, mg dm^−3^)	77 (53.5–108)	8 (4–17)	3.94e−12	−0.825	Large
Mn (DTPA, mg dm^−3^)	41.6 (12–99.2)	5.65 (3.35–13.1)	8.95e−07	−0.585	Large
Zn (DTPA, mg dm^−3^)	1.7 (0.8–3.65)	0.7 (0.475–0.925)	4.18e−08	−0.652	Large
B (mg dm^−3^)	0.2 (0.163–0.28)	0.14 (0.12–0.152)	5.26e−12	−0.814	Large
Al (cmolc dm^−3^)	0.09 (0.07–0.383)	0.21 (0.107–0.46)	0.0178	0.283	Small
Al saturation (m, %)	2.85 (2.1–12.1)	16.35 (10.3–29.2)	1.84e−06	0.568	Large
Potential acidity (H+Al, cmolc dm^−3^)	2.9 (2.12–4)	1.45 (1–2.12)	2.09e−07	−0.618	Large
Sand (%)	64.25 (58–70.5)	73 (53–83)	0.0074	0.32	Small
Silt (%)	7.5 (7.5–10)	2.5 (2.5–5)	6.54e−12	−0.763	Large
Clay (%)	27 (22–34.5)	23.25 (14.5–40.1)	0.0864	−0.206	Small
Clay activity (Tclay)	25.857 (19.5–28.1)	11.072 (9.33–12.9)	6.77e−11	−0.774	Large
ilr2: silt vs clay	-0.886 (− 1.07 to − 0.715)	-1.257 (− 1.54 to − 1.11)	5.49e−10	−0.735	Large
ilr1: sand vs (silt+clay)	1.208 (0.929–1.39)	1.789 (1.12–2.14)	5.80e−05	0.479	Large
K/T	4 (2.52–5.62)	2.7 (2.2–3.6)	0.0157	−0.29	Small
Mg/T	5.85 (4.38–7.92)	4.5 (3.3–5.7)	0.0258	−0.266	Small
Na/T	0.4 (0.4–0.5)	1 (0.7–1.2)	4.52e−11	0.778	Large
H+Al/T	49.75 (36.3–63.4)	56.65 (52.5–64.9)	0.0055	0.335	Medium
Ca+Mg/T	45 (32.6–58.9)	38.9 (30.5–42.8)	0.0074	−0.322	Small
Ca/T	39.8 (29.2–50.9)	32.9 (27.1–37.9)	0.0074	−0.321	Small
Mg/K	1.3 (0.825–1.9)	1.5 (1.1–2.2)	0.171	0.166	Small
Ca+Mg/K	11.5 (8.03–17.6)	12.9 (9.43–17.1)	0.272	0.134	Negligible
Ca/K	9.95 (7–15.2)	10.95 (8.1–15.2)	0.379	0.106	Negligible
Ca/Mg	6.75 (6.12–9.07)	7.25 (5.57–10)	0.91	0.017	Negligible

Between-group contrasts are given as FDR-adjusted q-values and Cliff’s δ (positive δ indicates higher values in tailing residues). Organic matter (OM) and total N in %; pH in water (1:2.5 soil:water); available P by Mehlich-1 (mg dm^−3^); exchangeable Ca, Mg and Al by 1 mol L^−1^ KCl and K and Na by Mehlich-1; potential acidity (H+Al) by pH 7.0 Ca-acetate buffer; sum of bases (SB), effective CEC (t) and potential CEC (T) in cmolc dm^−3^; base saturation (V) and Al saturation (m) in %; DTPA-extractable Fe, Mn, Zn and Cu in mg dm^−3^. Exchangeable cations and CEC are reported on a volume basis (cmolc dm^−3^), following the standard Brazilian routine protocol in which air-dried fine earth is dispensed by a calibrated volumetric scoop (Teixeira et al., 2017); division by the corresponding bulk density converts these to a mass basis (cmolc kg^−1^). Texture fractions are reported as sand, silt and clay (g kg^−1^) and as isometric log-ratio coordinates (ilr1, ilr2) for the compositional grain-size data

Compared with native soils, tailing residues are sand-enriched and silt-depleted, consistent with hydraulic reworking (Fig. 2a) that preferentially removes fine fractions (Williams, 2005). Organic matter and total nitrogen show the strongest lowering in residues, with median OM decreasing from 2.15 to 0.54% and total N decreasing from 0.18 to 0.03%. This shift is central because, in highly weathered tropical soils, organic matter contributes substantially to variable charge and to effective cation exchange capacity, so OM removal directly reduces nutrient retention and buffering (Oorts et al., 2003; Soares & Alleoni, 2008). In parallel, exchangeable bases, including Ca^2+^, Mg^2+^ and K^+^, collapse in residues and the sum of bases decreases from 3.13 to 0.95 cmol_c_ dm^−3^. Retention capacity also declines sharply, with effective CEC (t) falling from 3.23 to 1.29 cmol_c_ dm^−3^ and potential CEC (T) falling from 6.41 to 2.47 cmol_c_ dm^−3^, indicating that residues have a much lower capacity to retain cations and resist leaching.

Residues are also more acidic and more Al-constrained. Median pH (in H_2_O) decreases from 5.3 to 4.9 and Al saturation increases from 2.85 to 16.35%, consistent with the acidic tropical soil behavior where low base supply and low exchange capacity amplify Al stress (Kochian et al., 2004; von Uexküll & Mutert, 1995). H+Al is lower in residues, because OM and CEC are substantially smaller, so acidity effects are expressed more strongly per unit of exchange capacity. Micronutrient availability also declines systematically in tailing residues, with DTPA-extractable Fe, Mn, Zn, and Cu being lower than in native soils, since they become low-CEC, leaching-prone substrates.

**Fig. 6:**
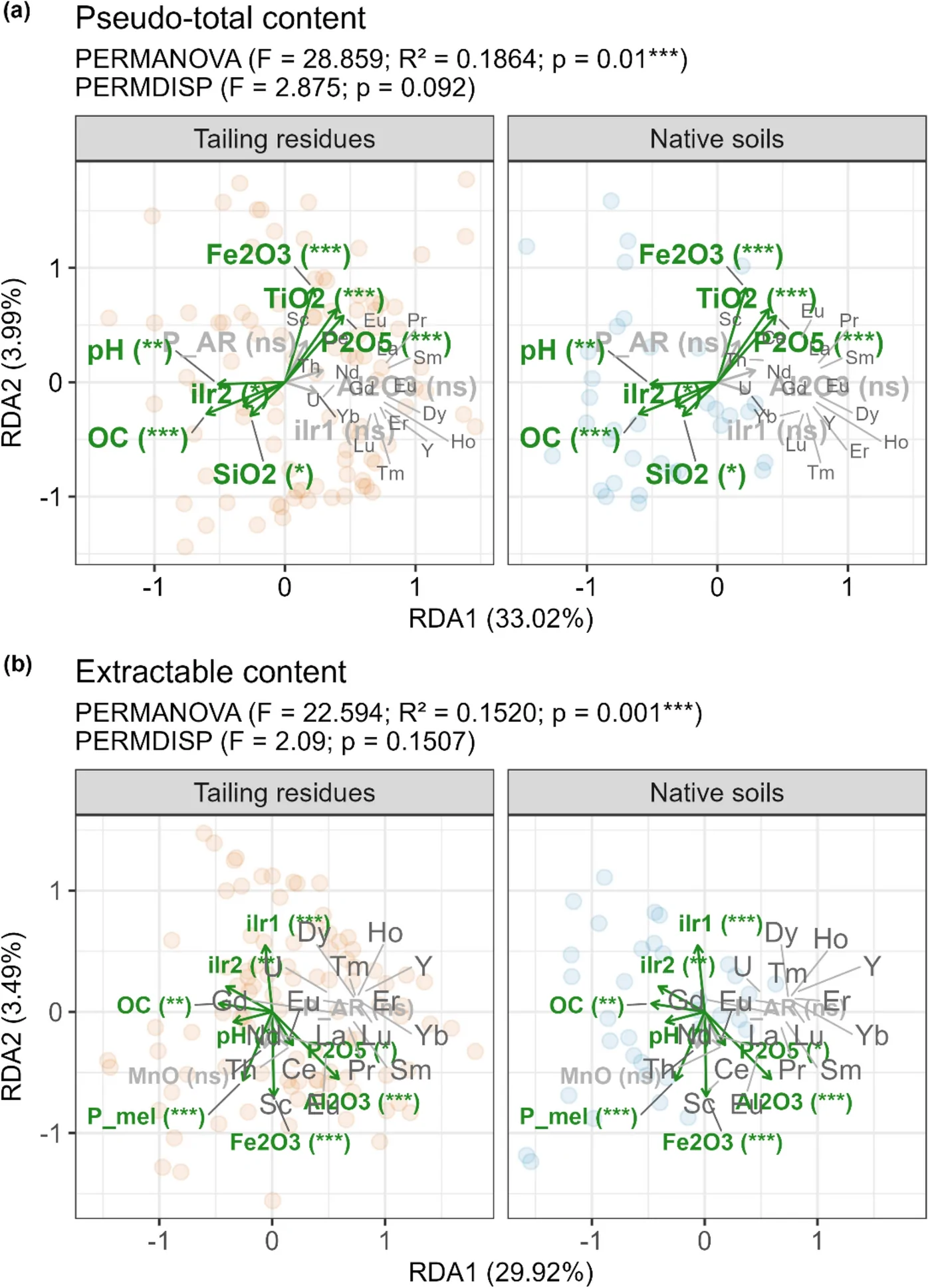
Redundancy analysis (RDA) showing the relationship between soil properties and **a** aqua regia (AR; pseudo-total) REE+Y, U, Th, and Sc, and **b** EDTA-extractable. Points represent individual samples from native soils and tailing residues. Green arrows indicate significant environmental variables, whereas gray arrows indicate non-significant variables. PERMANOVA and PERMDISP results are shown in each panel

RDA links these edaphic contrasts to the REE pools. For AR (Fig. 6a), the tailing and native groups differ significantly (PERMANOVA: R^2^ = 0.1864, *p* = 0.01), while dispersion does not differ at α = 0.05 (PERMDISP *p* = 0.092). The strongest environmental vectors aligned with the group separation include TiO_2_, P_2_O_5_, P_AR, and Fe_2_O_3_, whereas SiO_2_, OC and pH point in the opposite direction. However, REE+Y do not align strongly with these environmental vectors in the AR pool. This is consistent with REE+Y being concentrated in weathering-resistant accessory minerals (monazite, xenotime, zircon) that are only partially dissolved by aqua regia, so the pseudo-total signal primarily tracks heavy-mineral abundance rather than reactive geochemical phases. The dense-mineral proxies (TiO_2_, P_2_O_5_, Fe_2_O_3_) therefore distinguish residues from native soils but do not predict REE+Y at the variable level via AR digestion (Teixeira et al., 2005).

For the EDTA pool (Fig. 6b), the separation between native soils and tailing residues is also significant (PERMANOVA: R^2^ = 0.1520, *p* = 0.001), with no evidence of unequal dispersion (PERMDISP *p* = 0.1507). Unlike the AR pool, EDTA-extractable REE+Y align clearly with the Al_2_O_3_ and P_2_O_5_ vectors, indicating that their potentially extractable fraction is controlled by surface accessibility on aluminosilicate phases and by phosphate-mediated retention and release. Fine-fraction texture (ilr1, ilr2) and OC are also significant predictors, whereas pH is not significant in this model. Because OC and P_mel also reflect the strong contrast in fertility and fine-fraction abundance between native soils and tailing residues, these relationships are best interpreted as coherent covariation along the native-soil–tailing-residue gradient rather than as evidence of single-factor causality. U in the EDTA pool should be interpreted with caution because U(VI) can be strongly influenced by variable sorption to fresh Fe oxyhydroxides in near-surface materials (Waite et al., 1994).

### EDTA-extractable fractions and mobility-class distribution of U, Th, Sc, and REE in tailing residues and native soils

In tailing residues, the lowest mean EDTA-extractable fractions occurred for U (9.4%) and Th (9.3%), and the highest occurred among the heavy REE (Yb 25.0%, Lu 24.4%, Er 23.8%, Tm 23.7%), followed by Y (22.4%) and Eu (22.4%). Maximum values reached 87.2% for Ce and stayed above 70% for Dy, Ho, Er, Tm, Yb and Lu. In native soils, U remained the least extracted element on average (2.8%), noticeably lower than in tailings, while the highest mean values were recorded for Yb (28.0%), Tm (26.6%), Ho (26.5%), Er (26.4%) and Lu (26.4%). The contrast U-tailing (9.4%) vs U-native (2.8%) represents more than a threefold rise in operational mobility associated with mining, while Th shows the opposite trend (tailing 9.3% vs native 15.0%): in tailings, Th remains preferentially hosted by monazite that survived hydraulic reworking, whereas U is more easily mobilized by oxidative dissolution (Langmuir, 1978; Smedley & Kinniburgh, 2023).

**Fig. 7:**
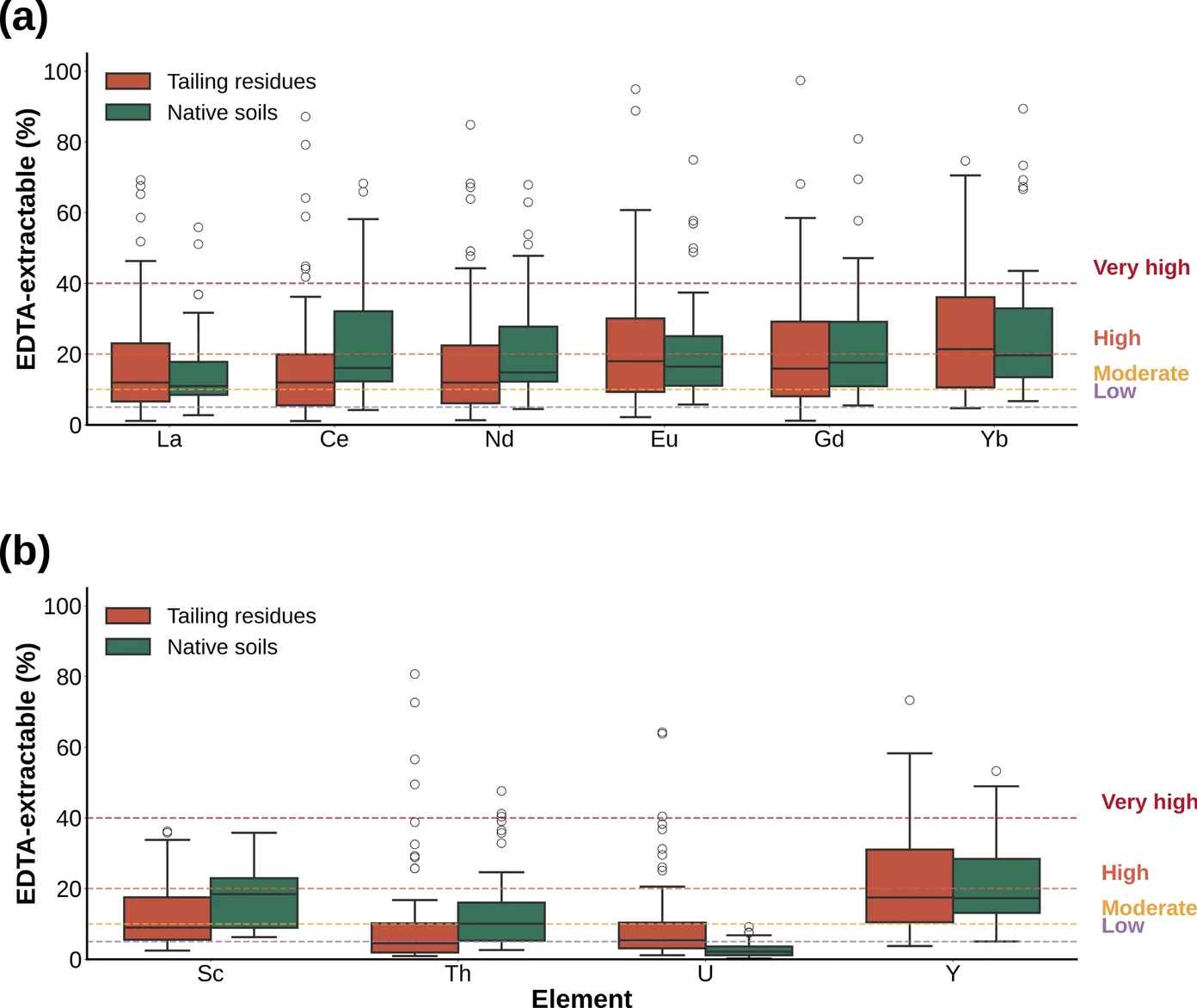
EDTA-extractable fractions (%) in tailing residues and native soils. **a** Representative REE: La, Ce, Nd, Eu, Gd, Yb. **b** Other trace elements: Sc, Th, U, Y. Dashed horizontal lines indicate EDTA mobility classes (Niesiobędzka, 2023): very low (< 5%), low (5–10%), moderate (10–20%), high (20–40%), and very high (> 40%). The remaining REE (Pr, Sm, Tb, Dy, Ho, Er, Tm, Lu) are shown in Supplementary Fig. 2. Boxplots show median, interquartile range, and outliers

In terms of the operational EDTA-mobility classes (Fig. 7a, b), U sits mostly in the ‘Very low’ to ‘Low’ classes in both environments, although the tailing distribution shifts noticeably toward the higher classes. Th follows the ‘Very low’/‘Low’ pattern in tailings (~ 55%) but distributes more evenly across ‘Moderate’ in native soils. Sc is predominantly ‘Low’ in tailings (mean 12.2%, median 9.0%) and ‘Moderate’ in native soils (mean 17.4%, median 18.5%), reflecting partial sequestration of Sc in secondary Fe/Mn-oxide-bound forms in native soils that is diluted by primary refractory phases in tailings (Babechuk et al., 2014).

Among the LREE, La and Pr fell predominantly in the ‘Moderate’ to ‘High’ classes in tailings, while Ce, Sm, and Nd displayed greater frequency within the ‘Moderate’ and ‘Low’ classes. In native soils, LREE are distributed mainly between ‘Moderate’ and ‘High’, particularly Ce, Nd, and Sm.

HREE and Y distributed predominantly within the ‘Moderate’ and ‘High’ classes in both environments (Fig. 7a, b). In tailing residues, Tb, Dy, Ho, Er, Tm, Yb, and Lu were mainly classified as ‘High’, with frequencies between 30 and 38%. In native soils, the HREE distributed across the ‘Moderate’ and ‘High’ classes: Yb, Lu, Tm, Ho and Er attain the highest means (> 25%), while Gd, Tb and Dy were more frequently ‘Moderate’.

Although the EDTA-based mobility index was originally proposed for Cu, Pb, and Zn by Niesiobędzka (2023), the conceptual approach based on the proportion of EDTA-extractable forms relative to total concentrations provides a useful framework for comparing potentially available fractions between native soils and tailing residues. In this study, this approach was adapted for REE, not as a formal speciation method, but as an operational comparison between EDTA-extractable and pseudo-total fractions. In an independent ion-adsorption REE tailing in southern China, Ou et al. (2022) reported that the exchangeable and carbonate-bound REE fractions increased while the Fe/Mn-oxide- and organic-matter-bound fractions decreased in less-disturbed soils; our EDTA results are consistent with that pattern, although the two studies concern different materials. This indicates that part of the REE pool in the native soil sits in forms less strongly retained by the soil matrix and is therefore more readily mobilized by the standardized EDTA extraction.

**Fig. 8:**
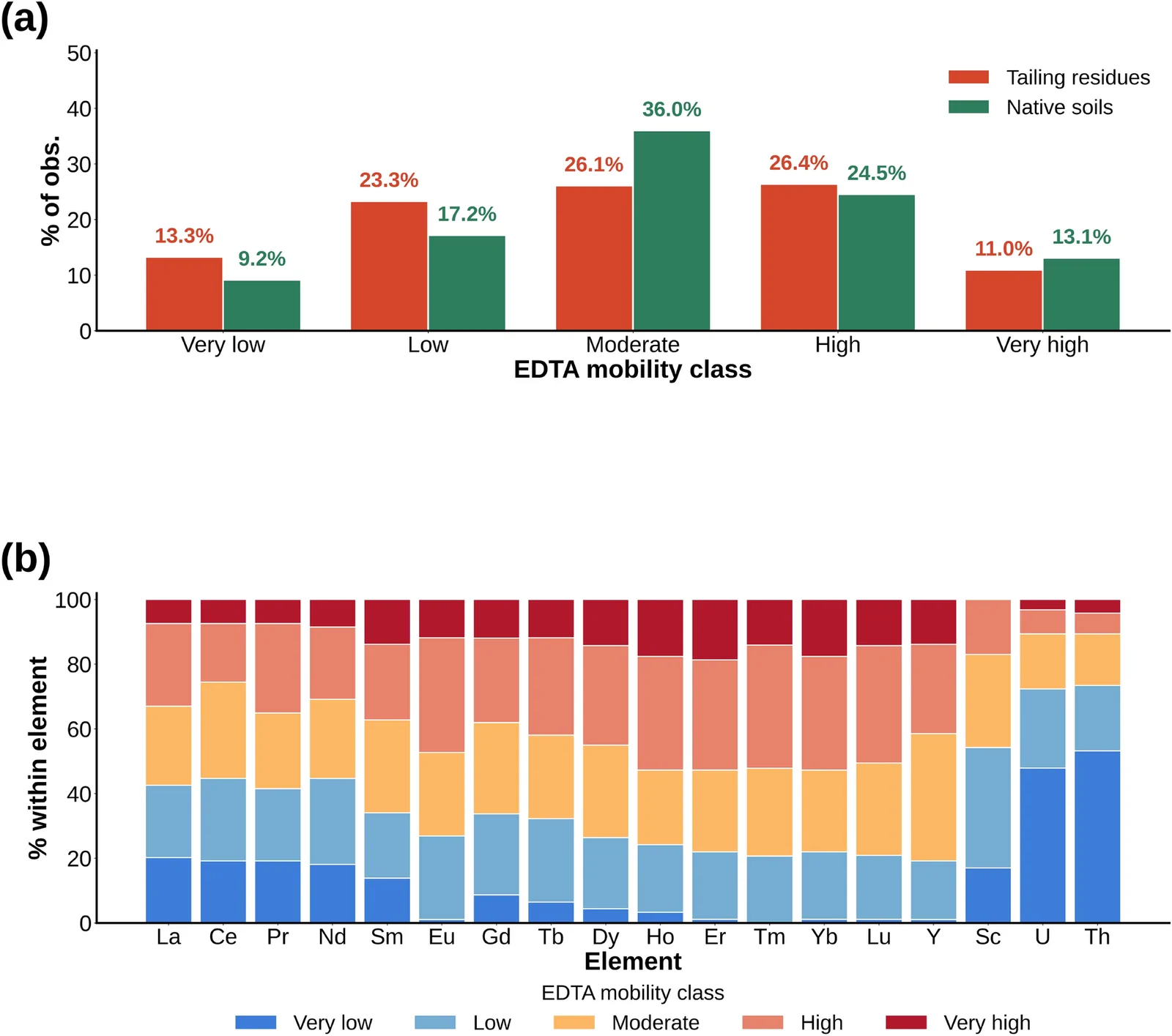
EDTA mobility class distribution. **a** Overall class distribution aggregated across the 18 elements (REE+Y, U, Th, Sc), comparing tailing residues with native soils. **b** Per-element stacked distribution within tailing residues, showing the share of each EDTA mobility class for every element. Class thresholds follow Niesiobędzka (2023): very low (< 5%), low (5–10%), moderate (10–20%), high (20–40%), and very high (> 40%)

Aggregating across all 18 elements (Fig. 8), the EMF class distribution in tailing residues was ‘Moderate’ (26.1%) and ‘High’ (26.4%) as the predominant classes, followed by ‘Low’ (23.3%), ‘Very low’ (13.3%) and ‘Very high’ (11.0%). In native soils, ‘Moderate’ clearly predominated (36.0%), followed by ‘High’ (24.5%), ‘Low’ (17.2%), ‘Very high’ (13.1%) and ‘Very low’ (9.2%). The ‘Moderate’+‘High’ combined share is 52.5% in tailings versus 60.5% in native soils, indicating that the operational mobility distribution shifts toward more variable (both lower and higher) extreme classes in tailings, whereas native soils retain a more concentrated ‘Moderate’ profile.

### Health and exposure implications

Three complementary indicators were computed on the filtered dataset (128 retained samples: 94 tailing residues and 34 native soils) to translate the geochemical and mobility results into screening-level metrics of human exposure and aquatic ecotoxicological pressure, all anchored on a lithology-aware geochemical baseline. The indicators are conservative and screening-grade; they support prioritization, not regulatory decision-making.

**Fig. 9:**
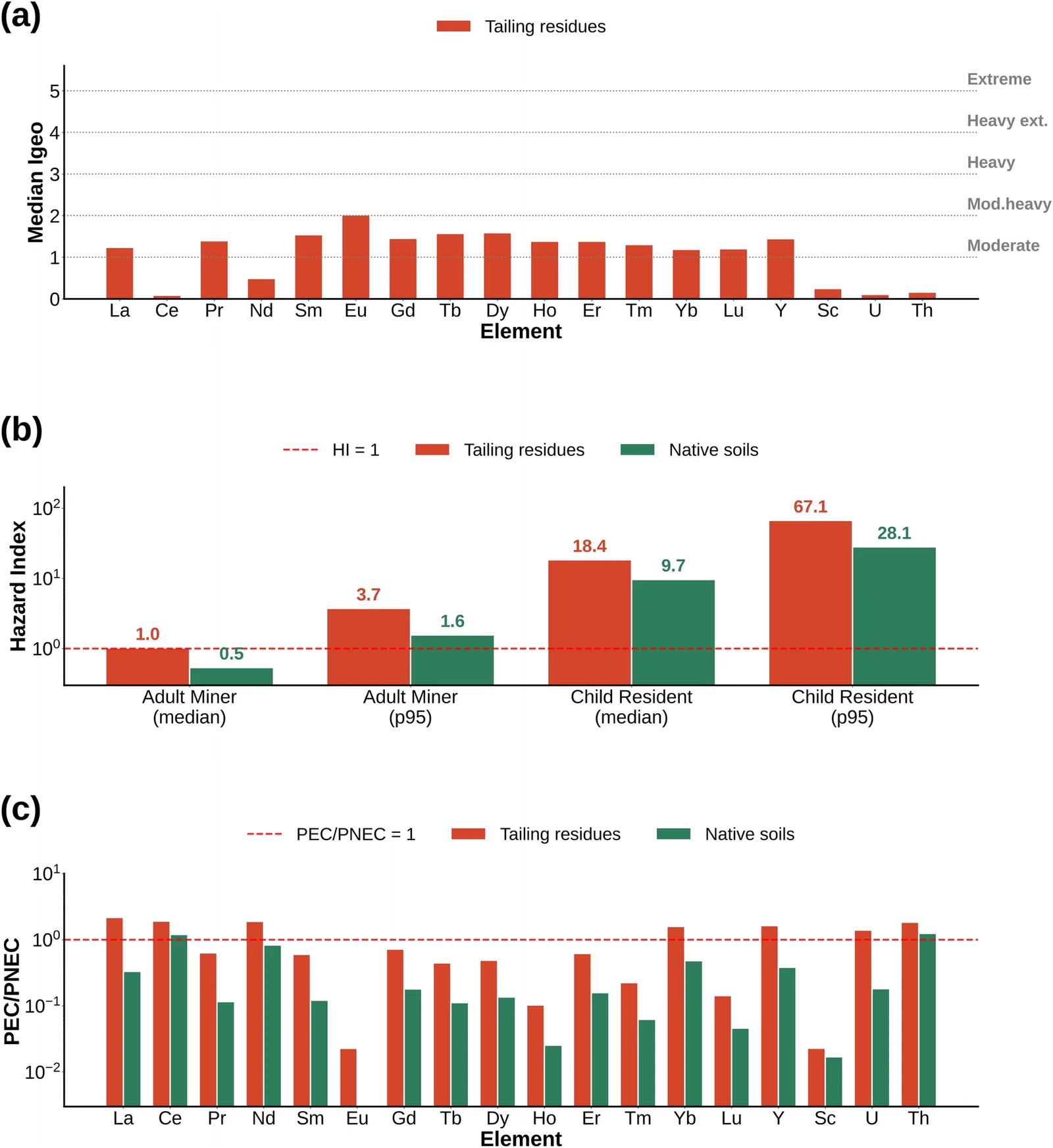
Contamination and exposure indicators by element and group. **a** Median geoaccumulation index (Igeo) per element, with class thresholds following Müller (1969); baseline = median of native soils. **b** Cumulative Hazard Index (HI = Σ HQ) for non-radiological exposure under the adult-miner and child-resident scenarios at median and 95th-percentile statistics; the dashed red line marks HI = 1. **c** Aquatic ecotoxicological screening PEC/PNEC for each element under the 95th-percentile scenario, using EDTA-extractable concentrations and literature soil–water Kd values. PNEC values for REE+Y from Sneller et al. (2000) and González et al. (2014), and from the British Columbia Ministry of Environment aquatic-life guideline for U. The dashed red line marks PEC/PNEC = 1

Using the median of the native soils as the local baseline (Müller, 1969), the median Igeo of tailing residues falls into the ‘moderately contaminated’ class (Igeo > 1) for the middle and heavy REE plus Y and Pr. Eu, Sm, Tb, Dy, Gd, Y, Pr, Ho, Er, Tm, La, Lu and Yb all sit between 1.19 and 2.02 at the median, with Eu the most enriched element on average. Sample-level maxima reach ‘extremely contaminated’ (Igeo > 5) for La (5.5), Pr (5.6), Sm (5.6), Eu (5.5) and Gd (5.3) in the most enriched residues, and exceed 4 (‘heavily contaminated’) for the remaining MREE-HREE and Y. U and Th are mostly in the lower classes at the median (Igeo ~ 0.1–0.2), consistent with their refractory hosts; even so, individual tailing samples reach Igeo = 2.3 (U) and 3.4 (Th), placing them in ‘moderately to heavily’ and ‘heavily contaminated’ classes respectively (Fig. 9a). As a robustness check, a Reimann-style anomaly threshold (median + 2 × mMAD of the native-soil population) flags essentially the same elements and tailing samples as the Igeo ‘moderately contaminated’ class (mean difference of about 5 percentage points, maximum 17 for Th; Supplementary Table 3, sheet ‘Igeo_robustness’), so the contamination pattern does not depend on the specific choice of reference.

Average daily doses (ADD) for soil ingestion, dust inhalation and dermal contact were estimated for two exposure scenarios (adult artisanal miner: body weight 70 kg, ingestion rate 100 mg d^−1^, inhalation rate 20 m^3^ d^−1^, exposed skin area 3300 cm^2^, soil adherence 0.2 mg cm^−2^, exposure frequency 180 d y^−1^, exposure duration 20 y; child resident: body weight 15 kg, ingestion rate 200 mg d^−1^, inhalation rate 10 m^3^ d^−1^, exposed skin area 2800 cm^2^, soil adherence 0.2 mg cm^−2^, exposure frequency 350 d y^−1^, exposure duration 6 y). Ingestion and inhalation followed the US-EPA framework (US-EPA, 1989, 1996), the dermal pathway followed RAGS Part E (US-EPA, 2004), and a particulate emission factor PEF = 1.36 × 10^9^ m^3^ kg^−1^ was used. Since dermal absorption factors have not been established for the REE, we applied a conservative default absorption fraction of 0.001; the dermal pathway added less than 1% to the Hazard Index in every scenario, as expected for poorly absorbed inorganic species. Oral reference doses for the REE were taken from Pang et al. (2002), and the US-EPA IRIS oral RfD was used for U and Th (3 × 10^−3^ and 5 × 10^−4^ mg kg^−1^ d^−1^). The cumulative Hazard Index (HI, summed over the three pathways) for tailings crosses 1 at both the median (HI ≈ 1.0 for the adult miner and 18 for the child resident) and the 95th percentile (HI ≈ 3.8 and 67) (Fig. 9b). Native soils stay below 1 for the adult miner at the median (HI ≈ 0.5) but not for the child resident (HI ≈ 10 at the median, 28 at the 95th percentile). Th (HQ ≈ 0.43) and Ce (HQ ≈ 0.27) dominate the tailings HI, followed by Nd (≈ 0.11), La (≈ 0.09) and Y (≈ 0.04). The child-resident scenario is the more sensitive of the two, driven by the higher soil ingestion rate and lower body weight, so Th and Ce are the elements to target first in any site-specific bioaccessibility work.

To screen the aquatic compartment, we coupled the EDTA-extractable concentration (taken as a proxy for the labile pool that could in principle reach porewater) to a literature-based soil–water partition coefficient (Kd; values from Sholkovitz, 1995 and the soil-specific Kd compiled in Mihajlovic & Rinklebe, 2018), and compared the resulting predicted environmental concentration (PEC) with Predicted No-Effect Concentrations (PNEC) for freshwater. PNEC values for the REE and Y were taken from the Dutch RIVM compilation (Sneller et al., 2000) and González et al. (2014). The U threshold was set at 8.5 µg L^−1^ (British Columbia Ministry of Environment aquatic-life guideline), and Th at 1 µg L^−1^ (conservative IAEA reference). For Sc the PNEC is poorly constrained (set at 10 µg L^−1^ here) and the resulting ratio should be treated qualitatively. In the 95th-percentile tailing scenario, PEC/PNEC exceeds unity for La (2.2 ×), Ce (1.9 ×), Nd (1.9 ×), Th (1.8 ×), Y (1.6 ×), Yb (1.6 ×) and U (1.4 ×). The remaining elements stay below the threshold (Fig. 9c). Native soils only marginally exceed unity for Ce and Th in the same scenario, so the residues, not the unaltered substrate, are the dominant source of potential aquatic hazard in the studied catchments. We note that the Kd values used here carry roughly one order of magnitude of uncertainty in tropical acidic systems (Mihajlovic & Rinklebe, 2018). The indicator is a screening proxy, not a quantitative risk estimate, and field measurement of dissolved REE in the impacted streams is the natural next step.

The U EDTA-extractable fraction rises from a mean of 2.8% (median 2.1%) in native soils to 9.4% (median 5.4%) in tailings, about a threefold increase in the operationally available pool. This shift is consistent with oxidative dissolution of U(IV) phases during alluvial reworking and subsequent partial (re)sorption to freshly precipitated Fe-oxyhydroxides under acidic to circum-neutral conditions (Smedley & Kinniburgh, 2023; Waite et al., 1994). Th, in contrast, shows the opposite trend (Tailing 9.3% vs Native 15.0%), in line with Th remaining mostly locked in detrital monazite (Williams et al., 2022). The combination of higher mobility and a PEC/PNEC of 1.4 × in the worst-case tailing scenario makes U, rather than Th, the priority actinide for downstream monitoring in this context, even though Th pseudo-totals are higher in absolute terms.

These indicators are screening-level and should be interpreted alongside the broader evidence. HQ and HI rely on default US-EPA exposure factors and uncertain oral RfD values for REE, while PEC/PNEC does not account for in-stream attenuation, organic complexation, or local Kd variability. The next steps are direct measurement of dissolved REE, U and Th in impacted waters, refinement of HQ using site-specific bioaccessibility tests, and analysis of bovine and fish tissues along Xingu tributaries to complete the soil–water–biota exposure pathway.

## Conclusion

The results demonstrate that REE enrichment in soils affected by illegal cassiterite-monazite mining cannot be interpreted independently from the lithological and mineralogical framework of the source rocks. In the Taboca district, the evolved A-type affinity of the granites of the Antônio Vicente batholith controls the natural distribution, fractionation, and partitioning of REE, Th, and U through accessory minerals such as monazite, xenotime, and zircon. The practical consequence is specific: in this granite-hosted Sn–REE system, a contamination assessment that ignores the monazite–xenotime–zircon inheritance would misread as anomalies the very high REE, Th and U values that are, in large part, geogenic; only the joint use of provenance normalization, mass-transfer and selective extraction separates the mining-added fraction from the inherited background.

This study demonstrates that the hydraulic reworking that generated the tailing residues selectively concentrates REE bearing heavy minerals while simultaneously degrading the physicochemical properties responsible for metal retention in tropical soils. The reduction in organic matter, cation exchange capacity, and fine reactive phases increases the operational mobility of HREE and Y, whereas U and Th remain predominantly associated with refractory mineral phases and Fe oxyhydroxides. These processes show that environmental risk in tropical mineralized systems is controlled not only by total concentration, but also by mineralogical hosts, soil degradation, and element mobility.

More broadly, the results highlight the need for geochemically contextualized approaches for environmental assessment in naturally enriched REE-Sn provinces, particularly given the absence of specific environmental guidelines for rare earth elements in Brazilian legislation. In systems characterized by strong lithological heterogeneity and mineralogical control, the integration of lithology-constrained baselines, REE fractionation systematics, and mass-transfer approaches provides a stronger framework for distinguishing geogenic signatures from mining-related concentration processes.

This framework can also inform environmental monitoring in REE-bearing provinces elsewhere, particularly in tropical regions with artisanal and illegal mining, where strong natural geochemical heterogeneity limits the use of generic contamination thresholds. For the Brazilian regulator (IBAMA and SEMAS-PA), the practical implications are threefold. First, lithology-aware geochemical baselines (such as the AVG-anchored, median-based local background derived here) should be adopted when setting reference values for soil, sediment or water in the Iriri-Xingu domain and analogous evolved A-type granite provinces. Second, U, Y and Yb combine comparatively high operational mobility with 95th-percentile PEC/PNEC ratios above unity and therefore deserve first priority in future monitoring; the other extractable HREE (Lu, Dy, Ho, Er and Tm) also warrant attention because their EDTA-extractable fractions are well above 20%, although their PEC/PNEC ratios remained below one in the scenarios screened here. Third, the present soil-based hazard index should be extended to the surface-water, sediment and biota compartments of the Xingu and its tributaries; the children resident in mining-impacted areas, for whom HI already exceeds 18 at the median, are the population that this extension should serve first.

## Supplementary Information

Below is the link to the electronic supplementary material.

Supplementary file1 (XLSX 11 KB)

Supplementary file2 (XLSX 15 KB)

Supplementary file3 (XLSX 93 KB)

Supplementary file4 (XLSX 13 KB)

Supplementary file5 (docx 756 KB)

## Data Availability

The datasets generated and analyzed during the current study are available in the Supplementary Information files, submitted with this manuscript. These include the geochemical, soil fertility, granulometric, EDTA-extractable, pseudo-total aqua regia, contamination-index, operational mobility, human-exposure, and ecotoxicological screening datasets used to support the results and interpretations.
