# Supplementary material for: Distribution, mobility and exposure risk of rare earth elements, uranium and thorium in soils impacted by illegal cassiterite–monazite mining in the southeastern Amazon, Brazil
Source: Environ Geochem Health. 2026 Aug 31;48(14):563. doi: 10.1007/s10653-026-03447-7 (PMC13529617; doi:10.1007/s10653-026-03447-7)
Supplement: Supplementary file 1 [file 10653_2026_3447_MOESM5_ESM.docx]

**Supplementary Figures**

**Supplementary Figure 1** Aqua regia (ng g^-1^) of trace elements and rare earth elements (REE) in tailing residues and native soil areas. Boxplots show median, interquartile range, and data dispersion, while points represent individual observations.

**Supplementary Figure 2** EDTA (ng g^-1^) of trace elements and rare earth elements (REE) in tailing residues and native soil areas. Boxplots show median, interquartile range, and data dispersion, while points represent individual observations.

**Supplementary Tables**

**Supplementary Table 1** Aqua regia (ng g⁻¹) variability for REE, U, Th and selected tracers in native soils derived from the Antônio Vicente Granite (AVG; n = 34) and in tailing residues (n = 96) from the Taboca district (southeastern Amazon), with published reference values for context. Values reported as the modified Median Absolute Deviation (mMAD = 1.4826 × MAD), a robust dispersion metric. Y is grouped with the HREE.

**Supplementary Table 2** Summary and between-group contrasts for AVG native soils (n = 34) and tailing residues (n = 96). Median and IQR are reported per group; mMAD is a robust dispersion metric (1.4826×MAD). p_MW is the two-sided Mann–Whitney U test p-value; q_FDR is the Benjamini–Hochberg FDR-adjusted q-value. Cliff’s delta is an effect size (positive = higher in tailing residues; negative = higher in native soils). Effect and Direction summarize magnitude and sign; Significant_FDR_0.05 flags q_FDR < 0.05. Texture fractions are compositional; ilr1 and ilr2 appear in 'dataset sheet' and were used for texture-aware analyses.

**Supplementary Table 3** Contamination, exposure and ecotoxicological index. Sheet contents: (i) Igeo per sample × element with Müller (1969) classification; (ii) Igeo summary by group with class frequencies; (iii) Hazard Quotient (HQ) per element, scenario (adult artisanal miner / child resident) and statistic (median / 95th percentile); (iv) cumulative Hazard Index (HI) summary; (v) PEC/PNEC for the aquatic compartment derived from EDTA-extractable concentrations and literature Kd; (vi) EDTA-extractable mobility-class frequencies (Niesiobędzka, 2023); (vii) whole-rock compositions of the six AVG varieties used for normalization (compiled from Teixeira et al., 2005). An additional sheet ('Igeo_robustness') compares the Igeo classification with a median + 2 × mMAD anomaly threshold as a robustness check.

**Supplementary Table 4** Analytical methods, detection/quantification limits and quality control (QA/QC). Compiled from the SGS GEOSOL and BIOAGRI certificates of analysis for the study samples.
